# *GDF9*^*His209GlnfsTer6/S428T*^ and *GDF9*^*Q321X/S428T*^ bi-allelic variants caused female subfertility with defective follicle enlargement

**DOI:** 10.1186/s12964-024-01616-8

**Published:** 2024-04-20

**Authors:** Yuwei Duan, Bing Cai, Jing Guo, Chen Wang, Qingyun Mai, Yan Xu, Yang Zeng, Yue Shi, Boyan Wang, Chenhui Ding, Minghui Chen, Canquan Zhou, Yanwen Xu

**Affiliations:** 1 Department of Gynecology & Obstetrics, Center for Reproductive Medicine, the First Affiliated Hospital, Sun Yat-Sen University, Guangzhou, Guangdong 510080 China; 2 Guangdong Provincial Key Laboratory of Reproductive Medicine, Guangzhou, Guangdong 510080 China; 3 Guangdong Provincial Clinical Research Center for obstetrical and gynecological diseases, Guangzhou, Guangdong 510080 China

**Keywords:** GDF9, Poor ovarian response, Follicular enlargement, Steroidogenesis, *In vivo* model

## Abstract

**Background:**

Antral follicles consist of an oocyte cumulus complex surrounding by somatic cells, including mural granulosa cells as the inner layer and theca cells as the outsider layer. The communications between oocytes and granulosa cells have been extensively explored in *in vitro* studies, however, the role of oocyte-derived factor GDF9 on *in vivo* antral follicle development remains elusive due to lack of an appropriate animal model. Clinically, the phenotype of *GDF9* variants needs to be determined.

**Methods:**

Whole-exome sequencing (WES) was performed on two unrelated infertile women characterized by an early rise of estradiol level and defect in follicle enlargement. Besides, WES data on 1,039 women undergoing ART treatment were collected. A *Gdf9*^*Q308X/S415T*^ mouse model was generated based on the variant found in one of the patients.

**Results:**

Two probands with bi-allelic *GDF9* variants (*GDF9*^*His209GlnfsTer6/S428T*^, *GDF9*^*Q321X/S428T*^) and eight *GDF9*^*S428T*^ heterozygotes with normal ovarian response were identified. *In vitro* experiments confirmed that these variants caused reduction of GDF9 secretion, and/or alleviation in BMP15 binding. *Gdf9*^*Q308X/S415T*^ mouse model was constructed, which recapitulated the phenotypes in probands with abnormal estrogen secretion and defected follicle enlargement. Further experiments in mouse model showed an earlier expression of STAR in small antral follicles and decreased proliferative capacity in large antral follicles. In addition, RNA sequencing of granulosa cells revealed the transcriptomic profiles related to defective follicle enlargement in the *Gdf9*^*Q308X/S415T*^ group. One of the downregulated genes, *P4HA2* (a collagen related gene), was found to be stimulated by GDF9 protein, which partly explained the phenotype of defective follicle enlargement.

**Conclusions:**

*GDF9* bi-allelic variants contributed to the defect in antral follicle development. Oocyte itself participated in the regulation of follicle development through GDF9 paracrine effect, highlighting the essential role of oocyte-derived factors on ovarian response.

**Supplementary Information:**

The online version contains supplementary material available at 10.1186/s12964-024-01616-8.

## Introduction

Infertility is affecting millions of people of reproductive age worldwide, with an estimated prevalence of 9%-12.5% [1–3]. Assisted reproductive technology (ART) is one of the most effective treatments for infertility. As the fundamental step of ART, controlled ovarian stimulation (COS) has been around for decades, however, clinicians still encounter the tricky situation of poor ovarian response (POR).

During COS, a cohort of antral follicles develop under gonadotropin (Gn) stimulation, with follicle size enlarged and estradiol level increased gradually. Antral follicles consist of an oocyte cumulus complex surrounding by somatic cells, including mural granulosa cells as the inner layer and theca cells as the outsider layer. Theoretically, genes mediating somatic cell response to Gn as well as oocyte-somatic cell interactions may be responsible for the development of POR [4, 5]. However, only few studies on variants in the *FSHR* [6, 7], *FMR1* [8, 9], and an *ESR1* [10], have been reported.

Growth differentiation factor (GDF), one of the members of TGF-β superfamily, is an oocyte-derived factor, secreted in oocytes throughout the entire period of follicle development [11, 12]. GDF9 plays an essential role in the folliculogenesis. GDF9 formed heterodimers with BMP15 activates Smad pathways, regulating the expression of AMH in both mouse primary granulosa cells and a human granulosa cell tumor-derived KGN cell line [13], and promoting proliferation of both rat and human primary granulosa cells [12, 14, 15]. Moreover, GDF-9 inhibited steroidogenesis by blocking 8-Br-cAMP-stimulated STAR expression in both human primary granulosa cells and theca cells [16]. Cumulus cells co-cultured with *Gdf9* dsRNA-injected oocytes exhibited limited expansion [17]. Collectively, GDF9 is involved in follicular development from the small to large antral follicle stage as well as ovulation. Female *Gdf9* knockout mice were sterile, and all follicles arrested at the primary follicle stage [18]. Thus, *in vivo* study on the role of GDF9 at the antral follicle stage is still lacking.

In human cases, several heterozygous variants in *GDF9* have been reported to be associated with primary ovarian insufficiency (POI), polycystic ovary syndrome (PCOS), and dizygotic twinning respectively [19], and one homozygous variant was reported to be associated with POI [20]. However, functional study of variants was lacking, with only one been validated *in vitro* [21], and the phenotype of GDF9 variants needs to be determined.

Here, we identified two bi-allelic variants in *GDF9* (*GDF9*^*His209GlnfsTer6/S428T*^ and *GDF9*^*Q321X/S428T*^) from two unrelated infertile women with an early rise of estradiol level and defect in follicle enlargement. We also identified eight *GDF9*^*S428T*^ heterozygotes with normal ovarian response among 1,039 infertile women. *In vitro* functional analyses confirmed that these variants led to changes in the protein expression and secretion. Pathogenicity of these variants was validated by the *Gdf9* variant mouse model, in which abnormal estrogen secretion and defected follicle enlargement were observed, recapitulating the phenotypes of the two probands.

## Methods

### Clinical samples

Two probands, individual P1 and P2, were involved in IVF/ICSI-ET procedure at the Reproductive Center of the First Affiliated Hospital of Sun Yat-sen University. Data on their clinical characteristics and outcomes were collected. Peripheral blood from the two probands and their parents and sisters was applied for whole-exome sequencing (WES) or Sanger sequencing. In addition, an in-house database containing WES data of 1,039 infertile women treated with IVF/ICSI-ET at the same reproductive center were used to determine the frequency of *GDF9* variants in a sporadic female infertility population. WES was performed as we previously described [22]. Sanger sequencing was performed to verify the candidate *GDF9* variants in other family members. The primers used are listed in Table S1. Follicular fluid and images of oocytes were collected from the first and second oocyte retrieval cycles of individual P1. Ovarian stimulation data, follicular fluid, and images of oocytes were also collected from control women. Inclusion criteria for the control group included IVF/ICSI-ET treatment, normal ovarian response, regular menstrual cycle, and age-matching with individual P1. Finally, six women were included with a mean age of 36 years, and 28 days of average cycle length, and 11 days of Gn (detail information was shown in Table S2). On the day of oocyte retrieval, follicular fluid was collected from the first follicle aspirated, which was 10-14 mm in diameter, and contamination with blood was carefully avoided.

This study was approved by the institutional ethics committee of the First Affiliated Hospital of Sun Yat-sen University.

### Expression plasmids and cell transfections

For protein expression and secretion experiments, human wild-type *GDF9*, *GDF9* variants (c.627_628del (p.His209GlnfsTer6), c.961C>T (p.Q321X), and c.1283G>C (p.S428T)), and wild-type *BMP15* cDNAs were subcloned into the M02 vector (FulenGen, China), respectively. For the immunoprecipitation and protein purification assay, His_10_ tags were added into N-terminal of the *GDF9* proregion according to previous studies [23, 24].

HEK293T cells were maintained in DMEM/high glucose (C11995500BT, Gibco, USA) supplemented with 10% FBS (FSP500, ExCell Bio, China) and penicillin/streptomycin (15140163, Gibco, USA). Cell transfection was performed using Lipo2000 (12566014, Invitrogen, USA) according to the instruction. Cells and supernatant were collected 48-72 h after transfection.

### *In vitro* cleavage assay

HEK293T cells in one well of a six-well plate were transfected with human wild-type *GDF9* and c.1283G>C (p.S428T) *GDF9*, respectively. And cells were treated with 10μM furin inhibitor II (hexa-D-arginine, D6R) (HY-P1028, MCE, USA) to inhibit the cleavage of GDF9 intracellularly for 48 hours. Cells were collected in lysis buffer (100mM HEPES, 0.5% NP40, 10% glycerol, 1mM DTT) and were lysed for 30 min at 4℃. After centrifugation, the supernatant containing protein was collected. 1/6 volume of the supernatant was mixed with reducing protein sample loading buffer (LT101S, EpiZyme, China) directly as a 0 h sample. 4/6 volume of the supernatant was added with 10U/ml furin enzyme (P8077, NEB), and the last 1/6 volume of the supernatant was added with 10U/ml furin enzyme and 50μM D6R. The last two supernatants were incubated at room temperature according to the manufacturer’s instruction. A portion of the supernatant with furin enzyme only was collected 10/30/60/120 minutes after incubation. The supernatant with both furin enzyme and D6R was collected 120 minutes after incubation. All these samples were boiled in reducing protein sample loading buffer for subsequent western blot. Both GDF9 precursor and mature GDF9 were detected by western blot. The relative gray value of mature GDF9/ (mature GDF9 + GDF9 precursor) was calculated to indicate the extent of GDF9 cleavage by furin. The experiment was repeated 3 times.

### Immunoprecipitation

HEK293T cells in four well of a six-well plate were co-transfected with wild-type *BMP15* and wild-type/mutant His_10_-*GDF9* (8g wild-type BMP15+8g wild-type His_10_-GDF9; 8g wild-type BMP15+8g c.1283G>C His_10_-GDF9; 8g wild-type BMP15 + 4g c.961C>T His_10_-GDF9+ 4g c.1283G>C His_10_-GDF9). Cells were lysed 48 hours after transfection using IP lysis buffer (87787, Thermo, USA) containing a protease-inhibitor cocktail (78425, Thermo, USA) for 30 min at 4℃. The protein concentration of each group of lysates was normalized to 1.5mg/ml. A portion of the lysates was used as the input. The remaining lysates were incubated with anti-Flag magnetic beads (B26102, Bimake, China) on a rocking platform overnight at 4℃, followed by magnetic separation. The immunoprecipitated complexes were washed with the PBST buffer three times, boiled in reducing protein sample loading buffer (LT101S, EpiZyme, China) for 10 min, and subjected to PAGE electrophoresis and western blotting after finally magnetic separation. Primary antibodies used were mouse anti-His-tag (1:1000, HA601079, Huabio, China) for detecting His_10_-GDF9, rabbit anti-BMP15 (1:2000, 18982-1-AP, Proteintech, China) for detecting BMP15 precursor. The experiment was repeated 3 times.

### Purification of recombinant human pro-GDF9-BMP15 complex

Expression and purification of recombinant proteins were performed according to a previous study [25] with some modifications. HEK293T cells were cotransfected with both an expression plasmid for human His_10_GDF9 of wild type or c.1283G>C (p.S428T) sequence, along with the expression plasmid for wild type human BMP15, as described above. Cells producing pro-His_10_GDF9-BMP15 complex were grown to near confluence, and growth medium was then replaced by production media [DMEM: F12 medium without penol-red (21041025, Gibco, USA), 0.02% BSA, 0.01% heparin (HY-17567, MCE, USA)]. After 48 h of incubation, the conditioned culture medium and cell lysate were collected. Lysis buffer contained 50mM NaH_2_PO_4_, 500mM NaCL, 10mM imidazole, 10% glycerol, 1% NP40, and 1mM PMSF, which was subsequently adjusted to pH 8.0. Production medium and cell lysate containing pro-His_10_GDF9-BMP15 complex were mixed and incubated with Ni-NTA resin (88221, Invitrogen, USA) on a rotary agitator at 4 °C for 2 h. The pro-His_10_GDF9-BMP15 complex was eluted using elution buffer (50mM NaH_2_PO_4_, 500mM NaCL, 300mM imidazole, 10% glycerol, pH 8.0). The eluates were concentrated using a centrifugal filter device (UFC900308, Millipore, Germany) centrifuging at 8000 rpm. BMP15 levels in eluates were detected by western blot, and levels of His_10_GDF9^WT^-BMP15 or His_10_GDF9^S428T^-BMP15 in the eluates were adjusted to the same level for subsequent *in vitro* granulosa cell intervention experiments. Every experiment was repeated 3 times.

### *In vitro* granulosa cell culture

Human primary luteinized granulosa cells (hGCs) were harvested from pre-ovulatory follicles of women undergoing IVF-ET according to previous study [26]. hGCs were cultured in DMEM/F12 medium (C11330500BT, Gibco, USA) containing 10% FBS and penicillin/streptomycin. To determine the effect of variants on protein activation of the smad pathway, hGCs were incubated with purified recombinant His_10_GDF9^WT^-BMP15 or His_10_GDF9^S428T^-BMP15 for 1 hour and then lysed to extract cellular protein. Levels of p-smad2 and smad2 were detected by western blot. To confirm the regulatory role of GDF9 on P4HA2, hGCs were incubated with 200ng/ml recombinant mature GDF9 (HY-P72425, MCE, USA) with or without 10μM SB431542 ( HY-10431, MCE, USA), an inhibitor of GDF9 receptor ALK5, for 24-48 hour and then RNA and protein were extracted, respectively. Every experiment was repeated 3 times.

### ELISA

GDF9 was measured in follicular fluid collected at the first oocyte retrieval from individual P1 and in follicular fluid from other six control women using an ELISA kit (CSB-E12925h, CUSABIO, China). All follicular fluid collected at the second oocyte retrieval from individual P1 had been used for western blot, so there was no remaining follicular fluid for ELISA. Wild-type, *Gdf9*^*S415T/S415T*^, and *Gdf9*^*Q308X/S415T*^ female mice at the age of 10-12 weeks were executed at the stage of proestrus and their serum were collected. Serum levels of estradiol were measured using an ELISA kit (KGE014, R&D, USA), according to the manufacturer’s instructions, with sensitivity of 12.1 pg/mL. Serum levels of testosterone were measured using an ELISA kit (ab285350, abcam, UK), according to the manufacturer’s instructions, with sensitivity of 0.1 ng/mL. Three mice were included in each group.

### Generation of *Gdf9* mutant mice

*Gdf9* (GenBank: NM_008110.2) mutant mice carrying variant [c.922C>T (p.Q308X); c.1244G>C (p.S415T)] analogous to the affected individual P1 [c.961C>T (p.Q321X); c.1283G>C (p.S428T)] were generated *via* CRISPR/ Cas9 technology by GemPharmatech (Jiangsu, China). Male and female mice of the *Gdf9*^*Q308X/wt*^ and *Gdf9*^*S415T/wt*^ genotype were mated to produce *Gdf9*^*Q308X/Q308X*^, *Gdf9*^*S415T/S415T*^ and *Gdf9*^*Q308X/S415T*^ mice. Genotype of pups was validated by PCR and sanger sequencing. This study was approved by the animal ethics committee of the First Affiliated Hospital of Sun Yat-sen University. sgRNA sequences and genomic primers are shown in Table S1.

### Estrous cycle evaluation

A cotton swab moistened with normal saline was rotated gently in the vagina. Then, the vaginal fluid was smeared on slide and stained with Giemsa solution (C0131, Beyotime, China). Morphology of vaginal detached cells was observed under the microscope to determine the different stages of the estrous cycle, including proestrus (P), estrus (E), metestrus (M), and diestrus (D) phases. Estrous cycles of 10-12 weeks old wild-type, *Gdf9*^*Q308X/Q308X*^, *Gdf9*^*S415T/S415T*^, and *Gdf9*^*Q308X/S415T*^ female mice were recorded for 12 days. Three mice were included in each group.

### Fertility test

Wild-type, *Gdf9*^*Q308X/Q308X*^, *Gdf9*^*S415T/S415T*^, and *Gdf9*^*Q308X/S415T*^ female mice were continuously mated with fertile wild-type male mice from the age of 8 weeks to 20 weeks (female: male = 2:1). The number of pups and litters was recorded. Three cages of mice were recorded in each group.

### Ovarian stimulation and COC/GC collection

Wild-type, *Gdf9*^*S415T/S415T*^, and *Gdf9*^*Q308X/S415T*^ female mice at the age of 10-12 weeks were injected i.p. with 10 IU PMSG (P9970, Solarbio, China) followed by 10 IU of human chorionic gonadotropin (HCG) (YZ-1817, Solarbio, China) after 48 hours. Cumulus-oocyte complexs (COC) were extracted from oviducts 12 hours after HCG injection. Images of COCs were collected by an optical microscope (IX51, Olympus, Japan). Three mice were included in each group.

Wild-type, *Gdf9*^*S415T/S415T*^, and *Gdf9*^*Q308X/S415T*^ female mice were killed 48 hours after PMSG stimulation and ovaries were removed for subsequent photography, paraffin embedding or granulosa cells separation. To separate granulosa cells, ovaries were placed in PBS containing 1% w/v type I collagenase and digested in 37°C for 15 minutes after puncturing the antral follicles with a 30G needle under the somatoscope. Digested granulosa cells were filtered out using a 40um strainer for subsequent experiments. For qPCR experiments, granulosa cells from both ovaries of one mouse of wild-type and *Gdf9*^*Q308X/S415T*^ groups were taken as one sample. There were three mice in each group. For the RNA-seq experiment, granulosa cells from ovaries of three mice of the same genotype (wild-type or *Gdf9*^*Q308X/S415T*^) and age were taken as one sample. Each group contains three samples, including nine mice.

### Histological analysis of ovaries and uteri

Ovaries were collected at the stage of proestrus or 48 hours after PMSG stimulation from 10-12 weeks old wild-type, *Gdf9*^*Q308X/Q308X*^, *Gdf9*^*S415T/S415T*^, and *Gdf9*^*Q308X/S415T*^ female mice (three mice per group). Uteri were collected from wild-type, *Gdf9*^*S415T/S415T*^, and *Gdf9*^*Q308X/S415T*^ female mice (three mice per group) at the stage of proestrus at 10-12 and 32-34 weeks of age, respectively. Ovaries and uteri were fixed in 4% paraformaldehyde, dehydrated in graded alcohol and xylene, and embedded in paraffin. Paraffin-embedded ovaries were serially sectioned at 5 μm thickness for subsequent hematoxylin and eosin (H&E) staining. For follicle-counting of ovaries collected at the stage of proestrus, every fifth ovary section was stained with H&E. According to the well accepted standards established by Pedersen and Peters [27], the primordial, primary, secondary, antral, and atretic follicles were identified and counted. In addition, the area of ovarian sections was measured by imageJ (version 1.51). Both data about follicle counts and data about follicle counts per area were calculated. To analyze the largest follicles in the ovaries collected 48 hours after PMSG stimulation, all consecutive sections were viewed under a microscope, and the section with the largest follicle located was finally stained with H&E. Images were scanned by an automatic digital pathology slide scanner (KF-PRO-020, Kfbio, China).

### Immunofluorescence and immunohistochemistry

Paraffin sections of the ovaries described above were also used for immunostaining. Paraffin sections were deparafinized in xylene 10 min 2 times followed by rehydration though a series of ethanol (100%, 100%, 90%, 80%, 70%) and ending with phosphate-buffered saline (PBS). For antigen retrieval, sections were heated in microwave (Midea, China) on medium-high level for 20 minutes with Tris–EDTA buffer (10 mM Tris, 1 mM EDTA solution, pH 9.0). After cooling down, slides were rinsed three times with PBS. For immunofluorescence, sections were blocked with 10% v/v goat serum (AR0009, BOSTER, China) for 30 min. Then, sections were incubated with primary antibodies overnight at 4℃ followed by 1 h with secondary antibodies, all diluted in blocking buffer. The primary antibody used was rabbit anti-STAR (1:250, 80751-1-RR, Proteintech, China). The secondary antibody used was Alexa Fluor 647 goat anti-rabbit IgG (1:500, ab150083, Abcam, UK). TUNEL-assay was performed by In Situ Cell Death Detection Kit (TMR red) (12156792910, Roche, Switzerland) according to the manufacturer’s instructions. Nuclei were stained with 4′,6-diamidino-2-phenyl-indole (DAPI, Beyotime, China). Images were captured with a fluorescence microscope (BX63, Olympus, Japan). For immunohistochemistry (IHC), sections were treated with 3% v/v H_2_O_2_ for 10 min and blocked using10% v/v goat serum (AR0009, BOSTER, China) for 10 min. Then, sections were incubated with primary antibodies overnight at 4℃. The remaining steps were performed using an IHC kit (KIT-9720, MXB, China), following the instructions. The primary antibodies used were rabbit anti-ki67 (1:500, 12202, CST, USA), mouse anti-P4HA2 (1:1000, 66604-1-IG, Proteintech, China). Images of were scanned with an automatic digital scanner (KF-PRO-020, Kfbio, China).

### Quantitative polymerase chain reaction

For human primary luteinized granulosa cells, total RNAs were extracted with RNAiso Plus reagents (9109, TaKaRa, Japan). For RNA extraction from whole mice ovaries, ovaries were placed in 1.5 ml centrifuge tubes and abraded by adding RNAiso Plus reagents and steel beads. cDNAs were synthesized with the PrimeScrip RT Master Mix (RR036A, TaKaRa, Japan) according to the manufacturer’s protocol. For RNA extraction from granulosa cells collected from ovaries, RNeasy Micro Kit (74004, Qiagen, Germany) was used. The RNA concentration was determined by Qubit RNA HS assay kit (Q32852, Thermo, USA). Because the total amount of RNA extracted from mouse granulosa cells was small, we used 5 ng of RNAs as input, which were reverse-transcribed and amplified using a single cell full-length mRNA amplification kit (N712, Vazyme, China) to obtain cDNAs. The quantitative polymerase chain reaction (qPCR) was performed with SYBR Green Pro Taq HS (RR820A, TaKaRa, Japan) and a real-time PCR system (QuantStudio 5, Applied Biosystems). We calculated relative mRNA levels by normalizing to GAPDH or *Gapdh*.

### Western blot

Follicular fluid was collected for western blot. *In vitro* cultured cells were lysed by RIPA buffer (P0013B, Beyotime, China) containing protease-inhibitor cocktail and phosphatase inhibitor cocktail (HY-K0022, MCE, USA) for 30 min at 4℃. Cell supernatant was filtered using a 0.22um filter (SLGPR33RB, Millipore, Germany). Then the filtrate of cell supernatant was concentrated using a centrifugal filter device (UFC900308, Millipore, Germany) centrifuging at 8000 rpm for 30 minutes. Protein concentrations of these samples were measured using the BCA Protein Assay Kit (23225, Thermo, USA). Lysates, concentrated supernatants, and follicular fluid were mixed with reduced protein sample loading buffer to achieve protein concentration of 2.5mg/ml, 1mg/ml and 2.5mg/ml, respectively, and boiled at 95℃ for 10 min. Proteins (total amount of protein per sample per assay: cell lysate 25μg, concentrated supernatant 35μg, follicular fluid 50μg) were resolved by SDS-PAGE and transferred to PVDF membrane (ISEQ00010, Millipore, Germany). Membranes were blocked with 5% v/v skimmed milk for 1 hour followed by incubating with primary antibodies overnight at 4℃. Then, membranes were washed and incubated with HRP-conjugated anti-rabbit IgG (1:5000, 7074, CST, USA) or HRP-conjugated anti-mouse IgG (1:5000, SA00001, Proteintech, China) for 1 hour at room temperature. Protein bands were detected by iBright imaging system (CL1500, Invitrogen). Levels of GAPDH were used as internal controls for cellular protein. Primary antibodies used were mouse anti-GAPDH (1:5000, HRP-60004, Proteintech, China), rabbit anti-GDF9 (1:1000, ab93892, abcam, UK) for detecting GDF9 precursor (51kDa) and mature GDF9 (15kDa) , rabbit anti-BMP15 (1:2000, 18982-1-AP, Proteintech, China) for detecting BMP15 precursor (calculated molecular weight: 45kDa, observed molecular weight according to the instruction manual: 50kDa), rabbit anti-BMP15 (1:2000, ab108413, abcam, UK) for detecting mature BMP15 (14kDa), rabbit anti-p-smad2 (1:2000, 18338T, CST, USA), rabbit anti- smad2 (1:2000, 5339T, CST, USA) and mouse anti-P4HA2 (1:1000, 66604-1-IG, Proteintech, China).

### RNA-sequencing and analysis

Granulosa cells were isolated from 10-12 weeks old mice, as described above. Total RNAs were extracted with RNAiso Plus reagents (9109, TaKaRa, Japan), and 1μg total RNA was used for following library preparation. Libraries with different indexes were sequenced on an Illumina HiSeq platform according to manufacturer’s instructions. Transcripts in fasta format were converted from known gff annotation file and indexed properly. Then, with the file as a reference gene file, HTSeq (v0.6.1) estimated gene and isoform expression levels from the pair-end clean data. Differential expression analysis was performed by the DESeq2 [28] package. Differentially expressed genes were defined as genes with adjusted *P* value < 0.05 and |log2FC| > 1. Gene set enrichment analysis (GSEA) was performed using Gene Ontology- Biological Process (GO-BP) gene sets and Reactome pathways obtained from the molecular signature database (MSigdb, http://www.gsea-msigdb.org/gsea/msigdb) by the clusterProfiler [29] package.

### Statistical analysis

Statistical analysis was performed with GraphPad Prism software (v9). Results were given as the means and standard deviations (SDs). Comparisons were made by two-tailed unpaired Student’s *t* tests when comparing two groups. ANOVA was performed when comparing more than two groups. When the *P* value of the ANOVA was significant, further multiple comparison tests were performed. Data were presented as mean±standard deviations (S.D.). *P* values of <0.05 were considered statistically significant.

## Results

### Clinical characteristics of the affected individuals

The age of individual P1 and P2 was 36 and 30 respectively. Both were diagnosed with primary infertility. In addition, individual P1 had a history of endometrial atypical hyperplasia when she was 33 years old. As shown in Table 1, normal karyotype was detected for both individuals. AMH value of P1 and P2 was 0.78 and 1.93 ng/ml respectively, and basal FSH level was 16.24 and 15.58 IU/L respectively. Individual P1 had a regular 25-days menstrual cycle at her young age and experienced an irregular menstrual cycle at the time of visit, mostly 35 days. The menstrual cycle of individual P2 was 24-25 days, however, no ovulation was observed by ultrasound in five consecutive menstrual cycles in previous year before she came to our clinic. Individual P1 and P2 had a history of infertility of 2.5 and 1.5 years, respectively, at the time of visit.

**Table 1:** Clinical characteristics of individuals with *GDF9* bi-allelic variants

	P1	P2
DNA mutation	c.961C>T / c.1283G>C	c.627_628del / c.1283G>C
Protein mutation	p.Q321X / p.S428T	p.His209GlnfsTer6 / p.S428T
Age (years)	36	30
Height/weight (cm/kg)	165/62	156/56
Karyotype	46,XX	46,XX
Diagnosis	Primary infertility Endometrial atypical hyperplasia	Primary infertility
Duration of infertility (year)	2.5	1.5
Menstrual cycle (Days)	25-35	24-25
AMH (ng/ml)	0.78	1.93
FSH/LH (mIU/ml)	16.24/8.81	15.58/5.52
AFC (bilateral)	5	9

**Fig. 1:**
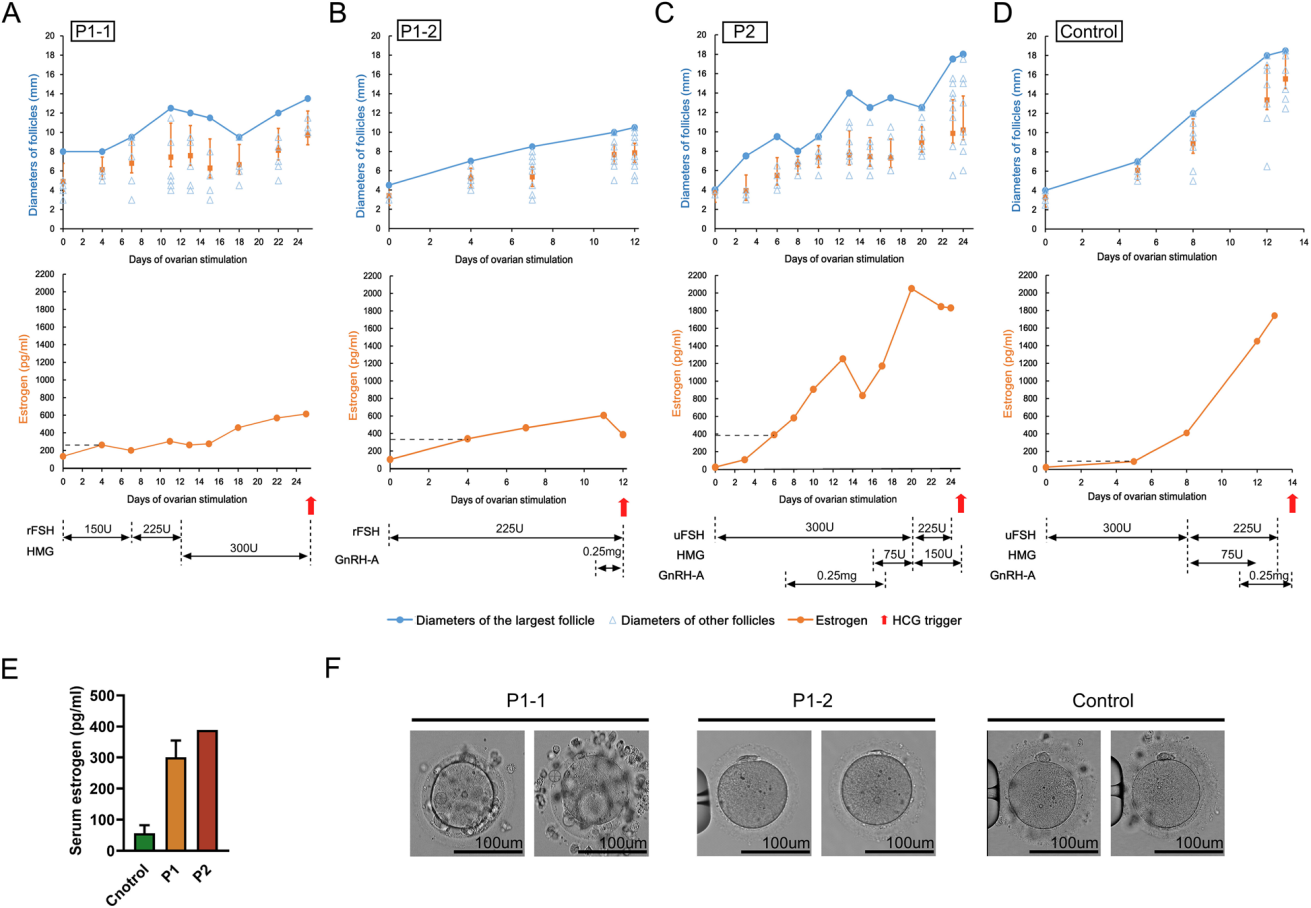
Ovarian stimulation process and oocytes of the affected individuals. **A** First ovarian stimulation cycle of individual P1. **B** Second ovarian stimulation cycle of individual P1. **C** First ovarian stimulation cycle of individual P2. **D** Ovarian stimulation cycle of control woman (numbered C1 in Table S1, in which her detail information was given) with normal ovarian response. A-D Changes in serum estrogen (the upper graph, orange line) and follicle diameter (the lower graph, each blue triangle represents each follicle, each orange vertical bar represents the standard deviation, and blue line represents the diameter of the largest follicle) during ovarian stimulation. The Y-axis of the upper graph represents follicle diameter, the Y-axis of the lower graph represents serum estrogen levels, and both X-axes represents days of ovarian stimulation. Dashed lines represent estrogen levels corresponding to ≥75% of follicles are 4-6 mm in diameter. Red arrow indicates the day of HCG trigger. Ovarian stimulation medication is shown below the picture and the numbers represent the daily dosage in the corresponding period. rFSH, recombinant human FSH (P1-1: Gonal-F, Merck Serono; P1-2: Puregon, Organon); uFSH, urinary human FSH (Urofollitropin, Livzon); HMG, human menopausal gonadotropin (Menotropins, Livzon); GnRH-A, GnRH antagonist (Cetrotide, Merck Serono); HCG, recombinant human chorionic gonadotropin (Ovitrelle, Merck Serono). E Serum estrogen levels in affected individuals (P1 and P2) and control women (n=6) during ovarian stimulation when ≥75% of follicles are 4-6 mm in diameter. F Pictures of oocytes obtained from control women and individual P1

Both patients entered IVF/ICSI treatment after more than three unsuccessful cycles of ovulation induction with clomiphene citrate or letrozole. In addition, individual P1 had an experience of 4 unsuccessful IVF treatment cycles in a local clinic, with no oocyte collected. Details of ovarian stimulation were not available. In the first IVF/ICSI cycle in our clinic, both patients experienced unexpected high estradiol level on stimulation Day 4-6 (when follicle sizes ranged from 4-6mm, estradiol levels reached 200-400pg/mL as shown in Fig. 1A-B, which was significantly higher than normal ovarian response women as shown in Fig. 1D, E), delayed follicle development with up to 24-25 days of Gn stimulation, and defects in follicular enlargement (Fig. 1A, B), especially in P1. After 25 days of Gn stimulation, all follicles of individual P1 did not meet the maturation criteria, and the largest follicle was only 13.5mm in diameter (Fig. 1A). Oocytes retrieved from individual P1 in the first IVF/ICSI cycle exhibited very poor quality with enlarged perivitelline space (PVS) and multiple irregular fragments in PVS (Fig. 1F). No insemination was performed. Different from individual P1, leading follicle size of individual P2 reached 18mm after 24 days of stimulation. However, under the same 300IU Gn dose per day, estradiol level dropped from 1,248 pg/ml on stimulation day 14 to 831pg/ml on stimulation day 16 when leading follicle size reached 14mm (Fig. 1B). Therefore, 75IU HMG was added for another 8 days. On the day of HCG triggered when leading follicles reached 18mm, only 3 out of 11 and 2 out of 13 follicles were larger than 14 mm in the right and left ovary respectively, corresponding to 1 and 8 oocytes retrieved in each ovary (Table 2). Oocytes from individual P2 were fertilized using conventional IVF method and the fertilization rate was normal compared to peers (Table 2). Considering the negative impact of long exposure of estradiol on endometrium, freeze all strategy was performed. After transfer of a frozen-thawed blastocyst, individual P2 became pregnant and finally gave birth to a healthy baby.

**Table 2:** IVF/ICSI outcomes of individuals with GDF9 bi-allelic variants

	P1-1		P1-2		P2	
Gn starting dose (IU)	150		225		300	
Days of Gn stimulation	25		13		24	
Total Gn dosage (IU)	6300		2925		8025	
E_2_ level on the day of hCG injection (pg/ml)	614		386		1822	
	L-OV	R-OV	L-OV	R-OV	L-OV	R-OV
Number of follicles at hCG injection						
d^a^ ≥18	0	0	0	0	0	1
15 ≤ d <18	0	0	0	0	2	2
10 ≤ d <15	1	3	2	1	3	0
5 ≤ d <10	2	1	6	4	8	8
Retrieved oocytes	0	3	3	2	8	1
Fertilization method	/		ICSI		IVF	
Fertilization rate (2PN)	/		2/5		8/9	
High-quality embryo rate (D3)	/		2/2		3/8	

*P1-1* the first therapy cycle of individual P1, *P1-2* the second therapy cycle of individual P1, *L-OV* left ovary, *R-OV* right ovary, *IVF* *in vitro* fertilization, *ICSI* intracytoplasmic sperm injection

^a^d= diameters

Individual P1 was a challenging case, and subsequent treatment was adjusted. Estradiol level was closely monitored during her second ovarian stimulation. When serum estradiol level reached 606 pg/mL after 12 days stimulation and a downward trend was suspected, HCG trigger was performed even though the maximum follicle diameter was only 10.5 mm (Fig. 1C). Four out of 5 oocytes obtained in this cycle were matured with normal morphology (Fig. 1F). Two high quality cleavage stage embryos from two normal fertilized oocytes were vitrified. Oral contraceptive pills were prescribed for one month before frozen-thawed embryo transfer, considering her history of endometrial atypical hyperplasia. Two frozen-thawed embryos were transferred in the subsequent programed cycle, leading to a singleton pregnancy. Finally, individual P1 gave birth to a healthy baby.

### Identification of *GDF9* bi-allelic variants

**Fig. 2:**
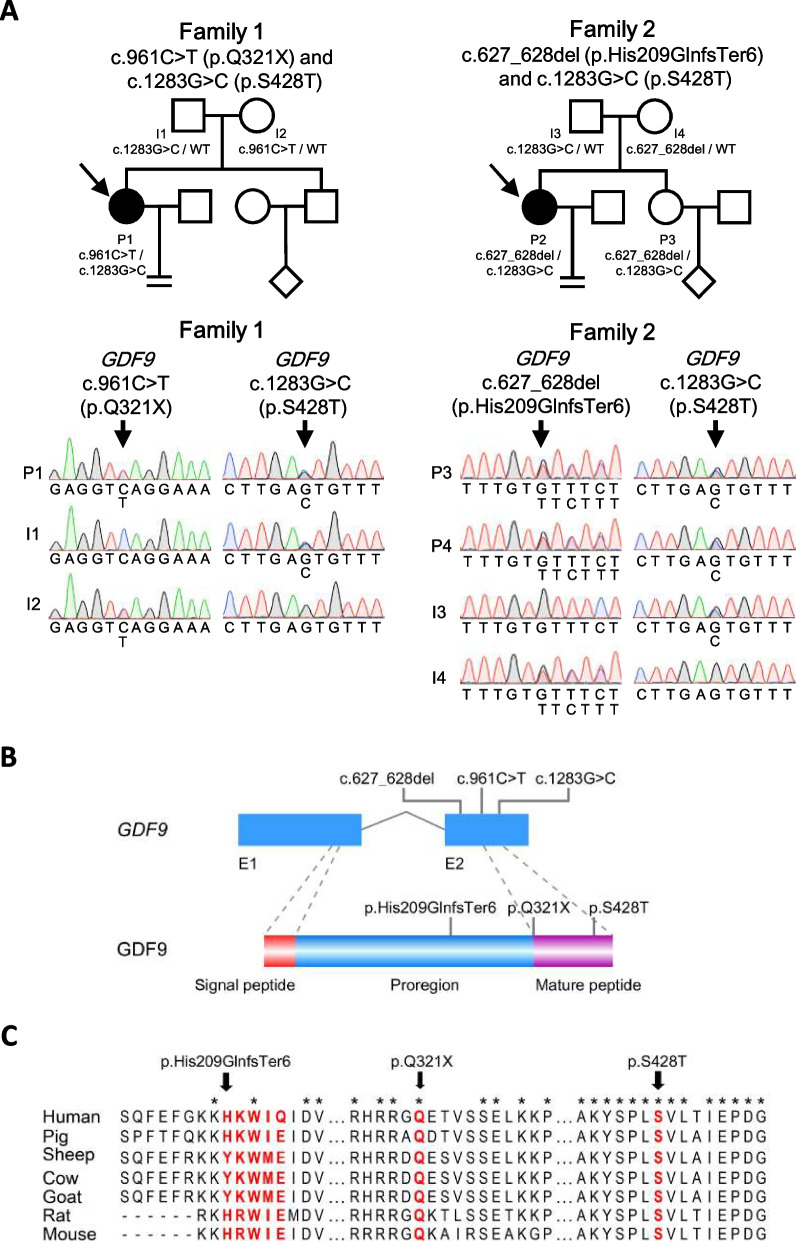
Identification of GDF9 bi-allelic variants. **A** Pedigrees of two affected families. Squares and circles represent males and females respectively. Diamonds represent members whose gender is unknown. Solid symbols indicate the affected members, and open symbols represent unaffected members. The equal sign indicates infertility. Arrows indicate probands. Sanger sequencing chromatograms of GDF9 are shown below. Downward arrows indicate the corresponding variants. **B** Schematic map of the variant positions in GDF9 at the genomic and protein levels. **C** Conservation of the affected amino acids is indicated by the alignment of seven species. Red letter represents amino acids affected by the variants. Asterisk indicates that the amino acid is conserved between species

Considering delayed follicle development with up to 24-25 days of Gn stimulation, and defects in follicular enlargement, whole-exome sequencing (WES) was performed on both individuals. It revealed that both individuals were carriers of *GDF9* (RefSeq accession number NM_005260.5) bi-allelic variants. Individual P1 carried a nonsense variant c.961C>T (p.Q321X) and a missense variant c.1283G>C (p.S428T). Sanger sequencing confirmed that her mother and father carried heterozygous c.961C>T (p.Q321X) variant and heterozygous c.1283G>C (p.S428T) variant respectively (Fig. 2A). Individual P2 carried the recurrent missense variant c.1283G>C (p.S428T) and a frameshift variant c.627_628del (p.His209GlnfsTer6). Sanger sequencing confirmed that both variants were inherited from father and mother respectively, and her younger sister (P3) carried the same genotype, who had a regular menstrual cycle, and gave a birth at age 27 (Fig. 2A).

We searched these three variants in the Genome Aggregation Database (gnomAD). Both the variant c.961C>T (p.Q321X) and c.627_628del (p.His209GlnfsTer6) were absent in the gnomAD, while the variant c.1283G>C (p.S428T) presented a low allele frequency, 0.00359. Furthermore, in our in-house database containing WES data of 1,039 infertile women treated with IVF/ICSI-ET, the variant c.961C>T (p.Q321X) and c.627_628del (p.His209GlnfsTer6) were not found, and the allele frequency of the variant c.1283G>C (p.S428T) was 0.0043, similar to the gnomAD. All women carried the variant c.1283G>C (p.S428T) were heterozygous, and none of them exhibited POI or POR at the time of presentation (Table S3).

From N-terminus to C-terminus, GDF9 consists of signal peptide, proregion, and mature peptide. Both the variant c.961C>T (p.Q321X) and c.627_628del (p.His209GlnfsTer6) result in the translation termination prior to the appearance of the mature peptide (Fig. 2B). The c.1283G>C (p.S428T) variant affects a residue at the C-terminal region, which is responsible for the binding of GDF9 to its receptor (Fig. 2B). In addition, amino acids affected by these variants are conserved across species (Fig. 2C). This evidence suggested that these variants may be pathogenic.

### Variants affect GDF9 expression and GDF9-BMP15 interactions

**Fig. 3:**
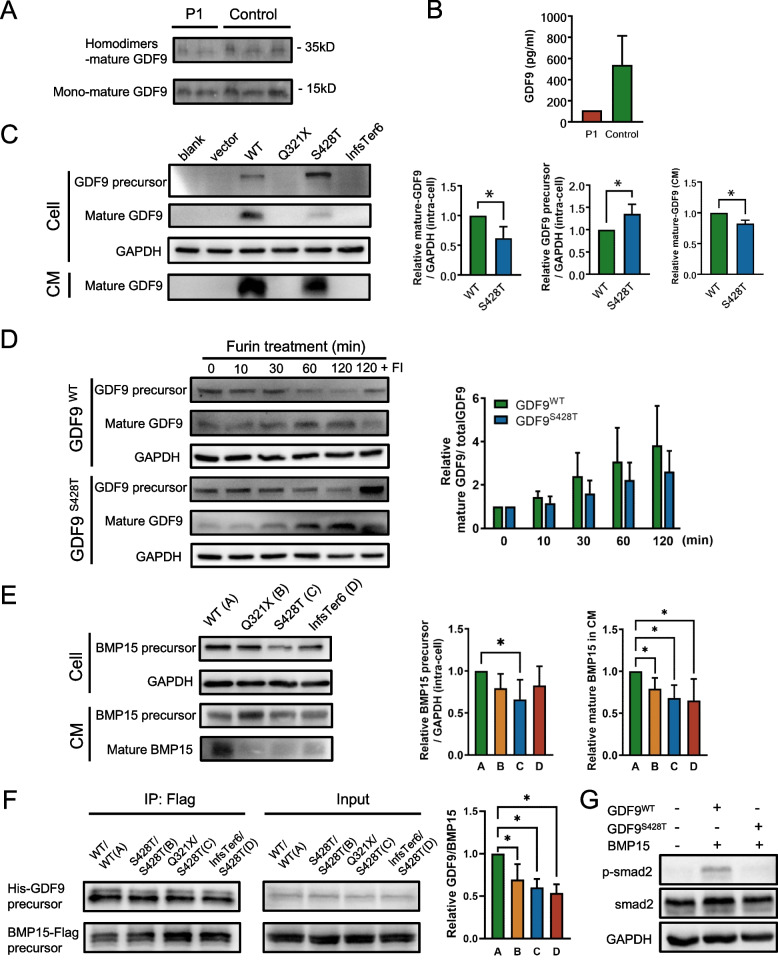
Effects of GDF9 variants on gene expression and GDF9-BMP15 interactions. **A** Western blots of mature GDF9 proteins expressed in follicular fluid collected from the first and second retrieval cycle of individual P1 and age-matched controls (*n*=3). **B** ELISA of GDF9 in follicular fluid collected from the first retrieval cycle of individual P1 and age-matched controls (*n*=6). **C** Western blots of GDF9 expression in HEK293T cells transfected with human *GDF9*^*WT*^, *GDF9*^*Q321X*^, *GDF9*^*S428T*^, and *GDF9*^*His209GlnfsTer6*^ respectively and their relative mature-GDF9 expression in culture medium (CM) (3 independent experiments). The amount of concentrated culture medium used for the detection was 35μl per sample per assay. lnfsTer6, His209GlnfsTer6. *P < 0.05. D Western blots of GDF9 precursors and mature GDF9 over time during *in vitro* cleavage assay (3 independent experiments). Total GDF9=GDF9 precursor+mature GDF9. E Western blots of BMP15 expression in HEK293T cells co-transfected with human *BMP15* and human *GDF9*^*WT*^, *GDF9*^*Q321X*^, *GDF9*^*S428T*^, and *GDF9*^*His209GlnfsTer6*^ respectively and their relative BMP15 precursor expression in CM (3 independent experiments). lnfsTer6, His209GlnfsTer6. *P < 0.05. F Co-IP assay of the binding of GDF9 proteins to BMP15 protein. lnfsTer6, His209GlnfsTer6. *P < 0.05. G Western blots of p-smad2 and smad2 expression in human primary luteinized granulosa cells treated with purified recombinant pro-GDF9^WT^-BMP15 and pro-GDF9^S428T^-BMP15 protein respectively (3 independent experiments)

To evaluate the functional effects of the identified *GDF9* variants, we firstly collected the follicular fluid of individual P1 in her first and second retrieval cycle in our clinic. The GDF9 levels were reduced in P1 follicular fluid as compared to women of the same age as controls (Fig. 3A, B). Next, we transfected WT and variant *GDF9* vector into HEK293T cells. Western blotting indicated that, both Q321X and His209GlnfsTer6 variants resulted in no detectable mature GDF9 intracellularly or in the cell supernatant (Fig. 3C). However, the S428T variant significantly increased GDF9 precursor expression intracellularly, and decreased mature GDF9 both intracellularly and in cell supernatants, compared to the WT (Fig. 3C). GDF9 precursor is able to be cleaved to mature GDF9 by proconvertases, such as furin [19]. To investigate whether S428T affected the efficiency of furin enzyme in processing GDF9, an *in vitro* cleavage assay was performed. Although not statistically significant, we did find a tendency for the decreased efficiency of furin enzyme during the processing of GDF9^S428T^ (Fig. 3D).

GDF9 forms heterodimers with BMP15 for biological functions [25]. To address whether *GDF9* variants affect GDF9-BMP15 heterodimers, we co-transfected the WT and variant *GDF9* vector with the WT *BMP15* vector into HEK293T cells. Western blot showed that, compared to the WT, all three *GDF9* variants significantly decreased mature BMP15 expression in the supernatant (Fig. 3E). Meanwhile, S428T variant also decreased intracellular BMP15 expression (Fig. 3E). Then we performed co-immunoprecipitation assay. Results indicated that the S428T variant significantly reduced the interaction between GDF9 and BMP15, whereas the Q321X and His209GlnfsTer6 variants appeared to exacerbate this effect (Fig. 3F).

To assess the effect of the S428T variant on GDF9 activity, we purified pro-GDF9-BMP15 complex for *in vitro* intervention in human primary luteinized granulosa cells. Western blotting showed that the phosphorylation of the classical downstream of GDF9, smad2, was significantly decreased in the GDF9^S428T^ +BMP15 group (Fig. 3G).

### *Gdf9* bi-allelic variants cause subfertility and disturbed estrous cycle

To confirm that the identified *GDF9* bi-allelic variants indeed caused reduced fecundity, we constructed *Gdf9* variant mouse model. Based on the results of the sequence alignment, the Q321X and S428T variant loci of the human *GDF9* correspond to the Q308X and S415T loci of the mouse *Gdf9*, respectively. Therefore, we constructed the following mouse models: *Gdf9*^*Q308X/Q308X*^, *Gdf9*^*S415T/S415T*^, and *Gdf9*^*Q308X/S415T*^.

**Fig. 4:**
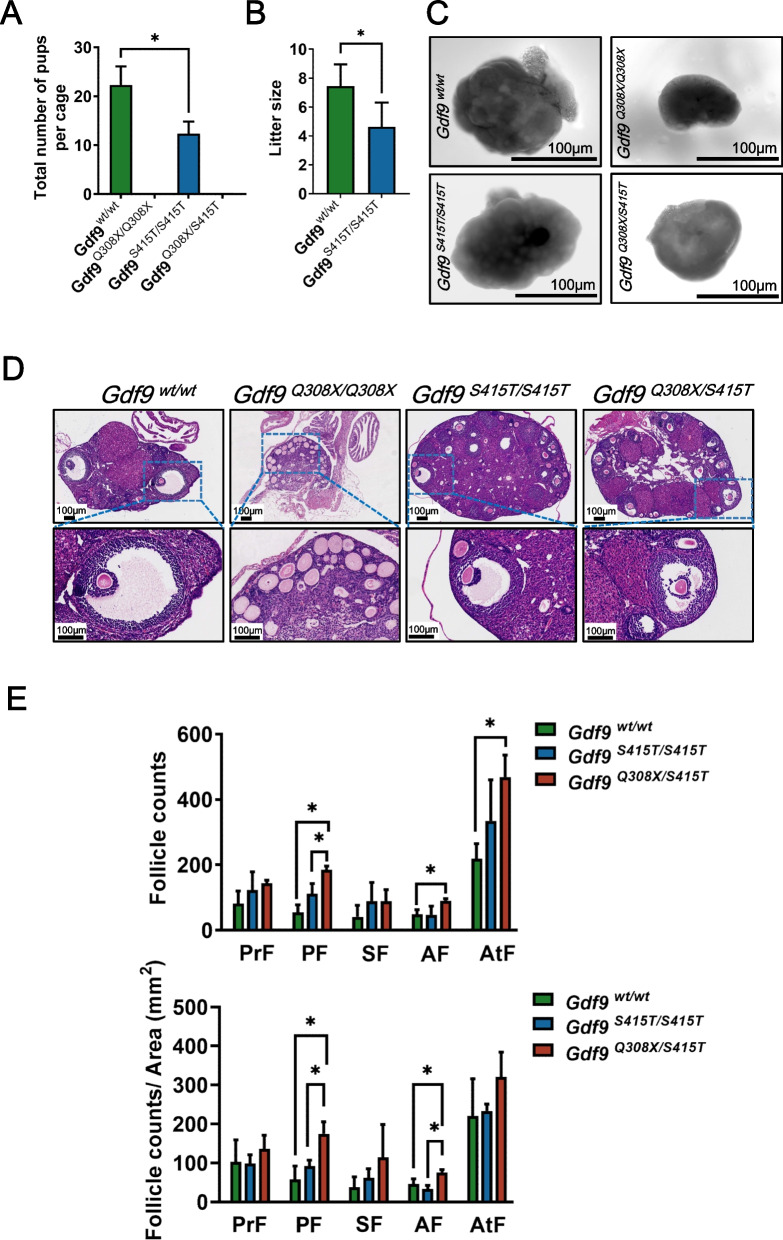
Effects of *Gdf9* variants on fertility and follicular development. **A** Average number of pups produced per cage (each cage contains 2 females and 1 male) by females *Gdf9*^*wt/wt*^ (*n*=3), *Gdf9*^*Q308X/Q308X*^ (*n*=3), *Gdf9*^*S415T/S415T*^ (*n*=3) and *Gdf9*^*Q308X/S415T*^ (*n*=3) when paired with wild-type males. *P < 0.05. **B** Average litter sizes of *Gdf9*^*wt/wt*^ *n*=9) and *Gdf9*^*S415T/S415T*^ (*n*=8) when paired with wild-type males. *P < 0.01. **C** Appearance of wild-type and variant ovaries as viewed through the stereoscope. **D** H&E staining of wild-type and mutant ovaries. **E** Follicle counts and follicle counts per area (mm^2^) of *Gdf9*^*wt/wt*^ (*n*=3), *Gdf9*^*S415T/S415T*^ (*n*=3) and *Gdf9*^*Q308X/S415T*^ (*n*=3) mice (PrF: Primordial follicles, PF: Primary follicle, SF: Secondary follicle, AF: Antral follicle, AtF: Atretic follicle). *P < 0.05

Mating of both *Gdf9*^*Q308X/Q308X*^ and *Gdf9*^*Q308X/S415T*^ females with wild-type males over a 3-month period failed to produce a pup. However, at the end of fertility assessment, when the mice were executed and dissected, we found one *Gdf9*^*Q308X/S415T*^ female pregnant with a near-mature fetus (Fig. S1). Both wild-type and *Gdf9*^*S415T/S415T*^ females were fertile. However, *Gdf9*^*S415T/S415T*^ females exhibited reduced fecundity as shown by lower number of total offspring and smaller litter size (Fig. 4A, B). Meanwhile, the fecundity of *Gdf9*^*Q308X/wt*^ females and *Gdf9*^*S415T/wt*^ females were the same as wild-types (Fig. S2 A, B). Daily analysis of vaginal smears revealed that estrous cycles of *Gdf9*^*Q308X/Q308X*^ and *Gdf9*^*Q308X/S415T*^ female mice were disturbed (Fig. S3 A). *Gdf9*^*Q308X/S415T*^ female mice continued to be in proestrus or estrus phase for up to 6 days, and had a significantly longer estrus phase than wild-type females (Fig. S3 B).

To determine if the reduced fecundity was associated with abnormal follicular development, we evaluated the morphology of ovaries. The appearance of the ovaries was first assessed. In the natural state, ovaries of wild-type and *Gdf9*^*S415T/S415T*^ mice were similar in size, while ovaries of *Gdf9*^*Q308X/Q308X*^ and *Gdf9*^*Q308X/S415T*^ mice were smaller (Fig. 4C). Next, the morphology and follicle count of ovaries were observed by H&E staining. All stages of follicles were found to be present in all groups, except for the *Gdf9*^*Q308X/Q308X*^ group in which only primordial and primary follicles were present in the ovaries (Fig. 4D). Total numbers of primary, antral, and atretic follicles, as well as the numbers of primary and antral follicles per area, were significantly higher in *Gdf9*^*Q308X/S415T*^ mice compared to wild-type mice (Fig. 4E). In addition, the number of primary and antral follicles per area in* Gdf9*^*S415T/S415T*^ mice was intermediate between wild-type and *Gdf9*^*Q308X/S415T*^ mice (Fig. 4E).

### ***Gdf9***^***Q308X/S415T***^ leads to abnormal antral follicle development through low proliferative capacity

**Fig. 5:**
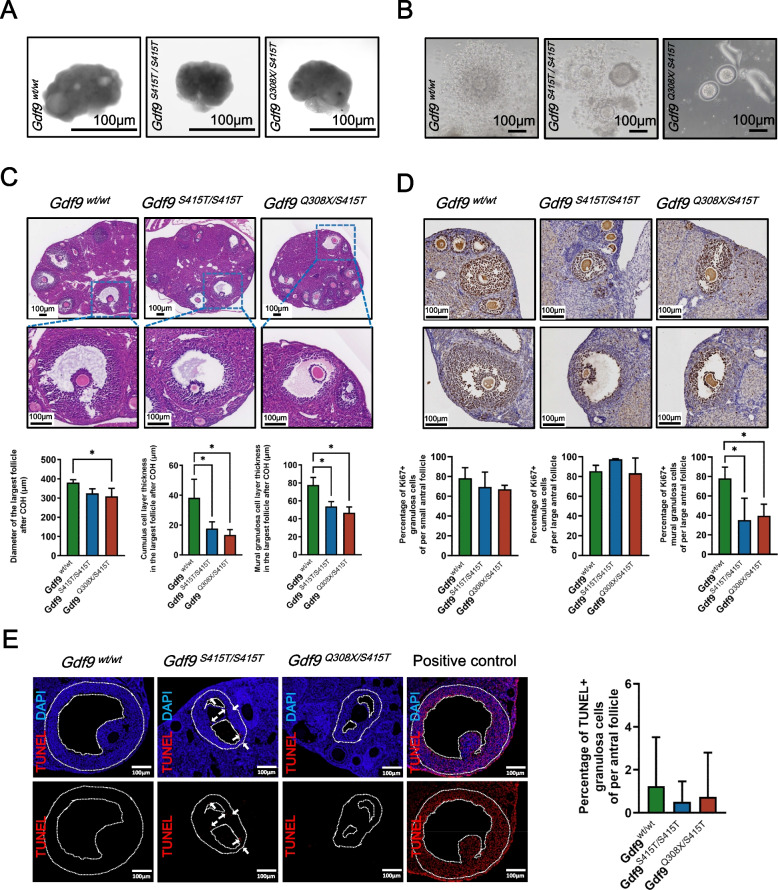
Effects of *Gdf9* variants on antral follicle development after PMSG stimulation. **A** Appearance of *Gdf9*^*wt/wt*^, *Gdf9*^*S415T/S415T*^ and *Gdf9*^*Q308X/S415T*^ ovaries 48 hours after PMSG stimulation as viewed through the stereoscope. **B** Appearance of cumulus-oocyte complex collected 12 hours after HCG injection. **C** H&E staining of *Gdf9*^*wt/wt*^ (*n*=3), *Gdf9*^*S415T/S415T*^ (*n*=3) and *Gdf9*^*Q308X/S415T*^ (*n*=3) ovaries 48 hours after of PMSG stimulation. The images shown were the sections where the largest follicle of each ovary was located. The follicle diameter, average thickness of the mural granulosa cell layer and cumulus cell layer were calculated for the largest follicle. *P < 0.05. D Ki-67 immunostaining in *Gdf9*^*wt/wt*^ (*n*=3), *Gdf9*^*S415T/S415T*^ (*n*=3) and *Gdf9*^*Q308X/S415T*^ (*n*=3) ovaries. The ki67 positivity rates of cumulus cells and mural granulosa cells in large follicles, and the ki67 positivity rates of granulosa cells in small follicles were calculated separately. *P < 0.05. E TUNEL (red) and DAPI (blue) immunofluorescence in *Gdf9*^*wt/wt*^ (*n*=3), *Gdf9*^*S415T/S415T*^ (*n*=3) and *Gdf9*^*Q308X/S415T*^ (*n*=3) ovaries. The dotted lines outline the boundaries of granulosa cells. Arrows indicate TUNEL-positive cells. TUNEL positivity rates of granulosa cells in each antral follicle were calculated

Human *GDF9* bi-allelic variants led to abnormal folliculogenesis, as evidenced by the failure of antral follicles to develop to mature size under prolonged Gn stimulation in individual P1 and P2. To verify this phenotype in animal model, we performed ovarian stimulation with PMSG. After 48 hours, there was little difference in the ovary size between the *Gdf9*^*S415T/S415T*^, *Gdf9*^*Q308X/S415T*^, and wild-type group (Fig. 5A). Although numbers of antral follicles were visible on the *Gdf9*^*Q308X/S415T*^ ovaries, sizes of antral follicles were smaller than that of wild-type (Fig. 5A). Next, ovaries were serially sectioned, and the section where the largest antral follicle located was selected for H&E staining. As shown in the results, the diameter of the largest antral follicle was significantly smaller and the thickness of the mural granulosa cell layer and cumulus cell layer were significantly thinner in *Gdf9*^*Q308X/S415T*^ mice compared with wild-type mice, and the data for *Gdf9*^*S415T/S415T*^ mice were intermediate between the above two groups (Fig. 5C).

COCs were collected 12 hours after HCG injection. The number of cumulus cells around the oocytes in the *Gdf9*^*S415T/S415T*^ group was significantly less than those in the wild-type group (Fig. 5B). Of note, oocytes of the *Gdf9*^*Q308X/S415T*^ group were nearly naked and only free granulosa cells could be seen in the buffer (Fig. 5B).

We then assessed the proliferative capacity and the degree of apoptosis of granulosa cells. The proliferative capacity was evaluated by immunohistochemical staining for ki67 (Fig. 5D). The proliferation index of granulosa cells in small antral follicles as well as mural granulosa cells and cumulus cells in large antral follicles were calculated (Fig. 5D). In granulosa cells in small antral follicles and cumulus cells in large antral follicles, the proliferative indices were similar in all groups. However, in the large antral follicles, the proliferative indices of mural granulosa cells in *Gdf9*^*S415T/S415T*^ and *Gdf9*^*Q308X/S415T*^ mice were significantly lower than that of wild-type mice. Next, the degree of apoptosis was observed by TUNEL staining (Fig. 5E). No significant differences were detected. These results implied that *GDF9* bi-allelic variant causes abnormal folliculogenesis by affecting the proliferative capacity of granulosa cells rather than apoptosis.

### ***Gdf9***^***Q308X/S415T***^ mice have abnormal estrogen secretion and developed atypical endometrial hyperplasia in old age

**Fig. 6:**
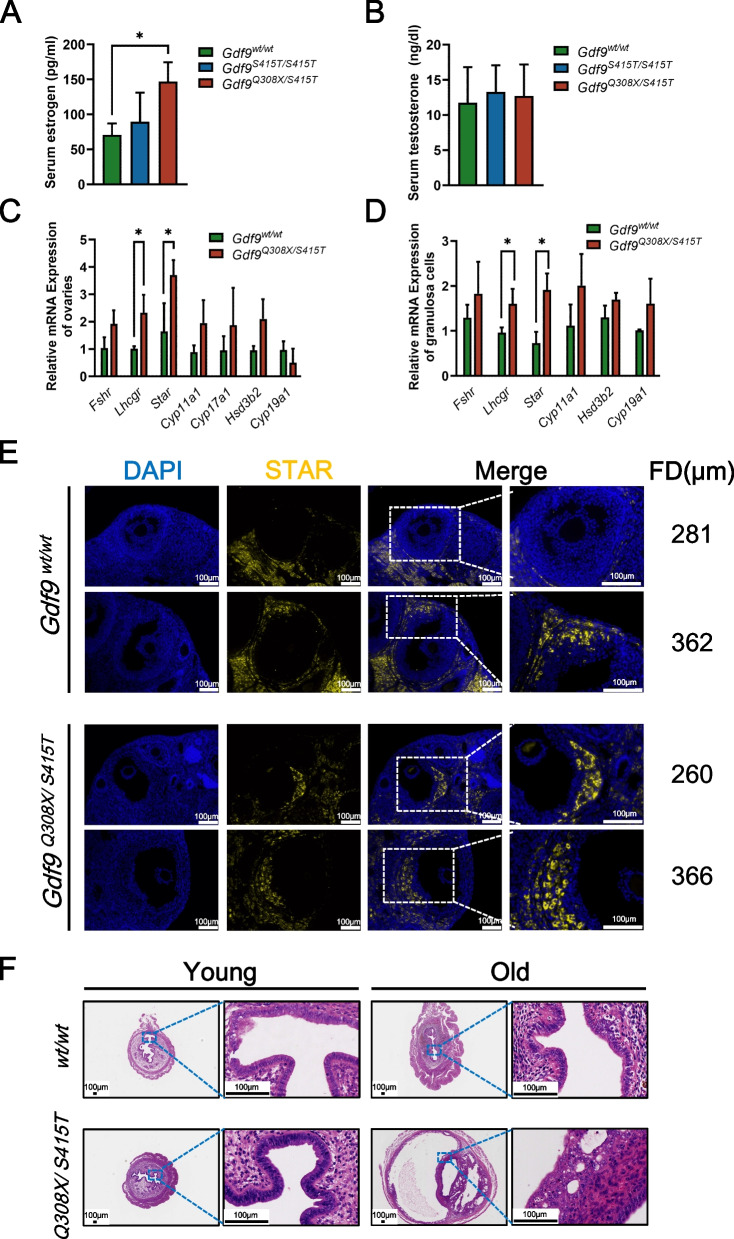
Abnormal estrogen secretion and atypical endometrial hyperplasia caused by *Gdf9* variants. **A** Serum estradiol levels in *Gdf9*^*wt/wt*^(*n*=3), *Gdf9*^*S415T/S415T*^ (*n*=3) and *Gdf9*^*Q308X/S415T*^ (*n*=3) mice at 10-12 weeks old during proestrus. *P < 0.05. **B** Serum testosterone levels in *Gdf9*^*wt/wt*^(*n*=3), *Gdf9*^*S415T/S415T*^ (*n*=3) and *Gdf9*^*Q308X/S415T*^ (*n*=3) mice at 10-12 weeks old during proestrus. **C** The mRNA expression of *Fshr*, *Lhcgr*, *Star*, *Cyp11a1*, *Cyp17a1*, *Hsd3b2*, and *Cyp19a1* of ovaries obtained from *Gdf9*^*wt/wt*^ (*n*=3) and *Gdf9*^*Q308X/S415T*^ (*n*=3) mice at 10-12 weeks old. *P < 0.05. **D** The mRNA expression of *Fshr*, *Lhcgr*, *Star*, *Cyp11a1*, *Hsd3b2*, and *Cyp19a1* of mouse granulosa cells collected 48 hours after PMSG stimulation. *P < 0.05. E STAR (yellow) and DAPI (blue) immunofluorescence in *Gdf9*^*wt/wt*^ and *Gdf9*^*Q308X/S415T*^ ovaries. Small (diameter ≤300μm) and large (diameter >300μm) follicles were shown respectively. FD, follicle diameter. F H&E staining of uteri obtained from young (10-12 week) and old (32-34 week) *Gdf9*^*wt/wt*^ and *Gdf9*^*Q308X/S415T*^mice at the stage of proestrus

Both individual P1 and P2 had an early rise in estrogen levels during the early days of ovarian stimulation. In another word, we observed unexpected high estradiol levels when follicle sizes were only 4-6mm, suggesting that *GDF9* bi-allelic variants may affect estrogen secretion. To confirm this phenomenon, we collected serum from mice in proestrus to detect estrogen. The result showed that estrogen levels of *Gdf9*^*Q308X/S415T*^ mice were higher than those of wild-type mice at the same phase (Fig. 6A), whereas serum testosterone levels were not significantly different (Fig. 6B). Next, qPCR was used to investigate the expression of gonadotropin receptors (FSHR and LHCGR) and ovarian steroidogenic key enzymes involved in estrogen synthesis (STAR, CYP11A1, CYP17A1, HSD3B2 and CYP19A1). Both *Lhcgr* and *Star* were upregulated in ovaries of *Gdf9*^*Q308X/S415T*^ mice (Fig. 6C). In addition, upregulation of *Lhcgr* and *Star* mRNA levels were confirmed in granulosa cells of *Gdf9*^*Q308X/S415T*^ mice (Fig. 6D). Further, earlier expression of STAR at the small antral follicle stage in *Gdf9*^*Q308X/S415T*^ mice was identified by immunofluorescence staining (Fig. 6D). Our results suggested that *GDF9* bi-allelic variants might bring estrogen secretion forward in small antral follicles by relieving its inhibition on STAR expression, which may explain the early elevation of estrogen in mouse mutation model and both probands.

As previously described, *Gdf9*^*Q308X/S415T*^ mice had a disturbed estrous cycle and could be in proestrus or estrus phase for consecutive days. It suggested that the endometrium of *Gdf9*^*Q308X/S415T*^ mice may be in an environment of high levels of estrogen. Meanwhile, individual P1 carrying *GDF9*^*Q321X/S428T*^ was diagnosed with atypical endothelial hyperplasia at the age of 33. To verify whether the *GDF9* bi-allelic variant can cause endometrial atypical hyperplasia, we collected uteri from 34-36 weeks old mice. HE staining confirmed that the uterus of *Gdf9*^*Q308X/S415T*^ mice showed atypical endometrial hyperplasia (Fig. 6E), manifested as cystic endometrial glands, papillary hyperplasia and nuclear atypia. However, no significant abnormalities were seen in the uteri of *Gdf9*^*Q308X/S415T*^ mice at 10-12 weeks (Fig. 6E).

### Altered gene expression profiles in granulosa cells of ***Gdf9***^***Q308X/S415T***^ mice at the antral follicle stage

**Fig. 7:**
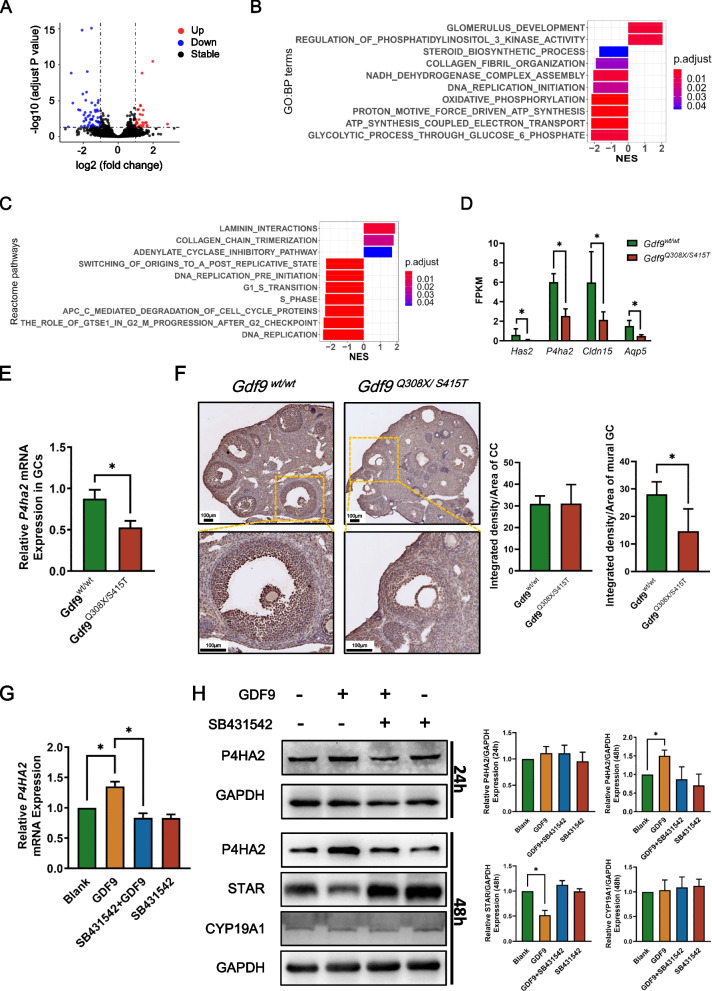
Altered gene expression in granulosa cells of *Gdf9*^*Q308X/S415T*^ mice explored by RNA sequencing. **A** Differentially expressed genes (DEGs) in granulosa cells collected from *Gdf9*^*wt/wt*^ and *Gdf9*^*Q308X/S415T*^ mice (three samples in each group, and each samples contains granulosa cells from three mice) 48 hours after PMSG stimulation. DEGs were defined as genes with adjusted *P* value < 0.05 and |log2FC|>1. B,C Enrichment analysis of GO-BP (Gene Ontology- Biological Process) terms (**B**) and Reactome pathways (**C**) using GSEA method. Normalized enrichment score (NES)>0 represent terms/pathways up-regulated in *Gdf9*^*Q308X/S415T*^ mice. NES<0 represent terms/pathways down-regulated in *Gdf9*^*Q308X/S415T*^ mice. D FPKM (Fragments Per Kilo bases per Million reads) of *Has2*, *P4ha2*, *Cldn15*, and *Aqp5* in granulosa cells collected from *Gdf9*^*wt/wt*^ and *Gdf9*^*Q308X/S415T*^ mice as a result of RNA-sequencing. *P < 0.05. E Expression of *P4ha2* in granulosa cells of *Gdf9*^*wt/wt*^ (*n*=3) and *Gdf9*^*Q308X/S415T*^ (*n*=3) mice after 48 hours of PMSG stimulation detected by qPCR. *P < 0.05. F P4HA2 immunostaining in ovaries of *Gdf9*^*wt/wt*^ and *Gdf9*^*Q308X/S415T*^ mice. The integrated density of P4HA2 staining of cumulus cells (CC) and mural granulosa cells (GC) were calculated separately. *P < 0.001. G mRNA levels of P4HA2 in human primary luteinized granulosa cells treated in vitro with GDF9 protein with or without 10μM SB431542 for 24 hours. SB-431542 is an inhibitor of ALK5, which is the receptor of GDF9. *P < 0.001. H Protein levels of P4HA2 in human primary luteinized granulosa cells treated in vitro with GDF9 protein with or without 10μM SB431542 for 24 hours and 48 hours. Meanwhile, protein levels of STAR and CYP19A1 after 48 hours treatment were shown. *P < 0.05

To explore the mechanism of the abnormal small follicle size after stimulation in *Gdf9*^*Q308X/S415T*^ mice, granulosa cells from wild-type and *Gdf9*^*Q308X/S415T*^ mice 48 hours after PMSG injection were collected for RNA sequencing. Differential analysis identified 27 up-regulated genes and 54 down-regulated genes in the *Gdf9*^*Q308X/S415T*^ group compared to the wild-type group (Fig. 7A). Subsequent GO enrichment analysis (Fig. 7B, Table S4) suggested that the highest absolute normalized enrichment score (|NES|) was the Glycolytic process through Glucose-6 Phosphate, which was significantly down-regulated in the *Gdf9*^*Q308X/S415T*^ group. And ATP synthesis, oxidative phosphorylation, DNA replication, and collagen fiber organization were also found significantly down-regulated. Meanwhile, regulation of phosphatidylinositol 3 kinase (PI3K) activity was significantly up-regulated in the *Gdf9*^*Q308X/S415T*^ group. Consistently, REACTOME enrichment analysis (Fig. 7C, Table S5) confirmed that DNA replication, cell cycle related pathways, and glucose metabolism were down-regulated in the *Gdf9*^*Q308X/S415T*^ group. In addition, laminin interactions and adenylate cyclase inhibitory pathway were up-regulated.

Next, genes related to antral follicular enlargement were analyzed, particularly genes involved in osmotic potential of follicular fluid (genes responsible for biosynthesis and modifying of versican, hyaluronan, proteoglycan, and collagen, such as *Vcan*, *Has2*, *Hspg2* and *P4ha1-3* ) [30, 31], in cell junctions (*Ocln*, *Tjp1* and the claudin family) [31], and in active water transport (the aquaporin family) [31]. We found that *Has2*, *P4ha2*, *Cldn15*, and *Aqp5* were significantly downregulated in the *Gdf9*^*Q308X/S415T*^ group (Fig. 7D).

Considering *P4ha2* is a collagen post-translational modification related gene involved in collagen fiber organization, and the expression level of *P4ha2* increases in human granulosa cells from small antral follicles to preovulatory follicles according to the GEO dataset GSE107746, which included transcriptome profiles of granulosa cells from five follicle development stages [32] (Fig. S4), we speculated that *P4ha2* may be one of the most important genes involved in size enlargement of antral follicles. To confirm the effect of GDF9 variants on P4HA2 expression, we detected *P4ha2* mRNA in granulosa cells separated from ovaries of wild-type and *Gdf9*^*Q308X/S415T*^ mice by qPCR. At the mRNA level, *P4ha2* was significantly reduced in *Gdf9*^*Q308X/S415T*^ mice (Fig. 7E). Immunohistochemistry was also performed to further clarify the protein expression of P4HA2 in mural granulosa cells and cumulus cells. The results showed that P4HA2 was significantly reduced in mural granulosa cells of *Gdf9*^*Q308X/S415T*^ mice compared with the wild-type mice, whereas there was no significant difference in cumulus cells (Fig. 7F). Further, we performed *in vitro* experiments on human primary luteinized granulosa cells to evaluate whether stimulation with GDF9 protein affects P4HA2 expression *in vitro*. Both qPCR and western blotting showed that GDF9 promoted P4HA2 expression in granulosa cells, which can be blocked by SB431542, an inhibitor of the GDF9 receptor ALK5 (Fig. 7G, H). As a reference, the effects of these treatments on STAR and CYP19A1 expression were shown together (Fig. 7H).

## Discussion

In the present study, we identified two bi-allelic genotypes consisting of three *GDF9* variants. These variants resulted in reduced secretion of GDF9, as well as weaker binding of GDF9 and BMP15 (observed in human cells), which in turn caused diminished granulosa cell proliferation, early rise in estrogen secretion, and defect in follicle enlargement (confirmed in the mouse model), ultimately leading to abnormal follicular development (observed both in human and mice). Our study emphasized the essential role of oocyte-derived factors in the regulation of follicular development.

GDF9 precursor forms mature GDF9 after removal of the proregion. In previous studies, *GDF9* variants were found associated with POI [33–37], diminished ovarian reserve [20, 21], and PCOS [38, 39]. A frameshift variant in the proregion was demonstrated leading to deletion of the mature GDF9 [20]. The rest were missense variants in the proregion [39]. In the present study, c.961C>T (p.Q321X) and c.627_628del (p.His209GlnfsTer6) were presented, two variants that have not been previously reported. The former was a nonsense variant at the junction of the proregion and the mature region, and the latter was a frameshift variant in the proregion. Theoretically, these two variants result in loss of GDF9 function due to failure to form the mature region of GDF9. It was confirmed in the *Gdf9*^*Q308X/Q308X*^ mice in our study, which displayed the phenotype with follicle growth ceased at the primary stage, consistent with that of *Gdf9* knockout mice. Missense variant, c.1283G>C (p.S428T), occurs in the mature region near the C-terminus. Interestingly, it was previously reported to be associated with PCOS [39, 40]. It led to a significant decrease in GDF9 secretion [39], which was consistent with our study. Mechanically, our results demonstrated that S428T may affect GDF9 secretion by interfering the post-translation processing of GDF9 through furin enzyme. In addition, we confirmed that S428T affected the formation of GDF9-BMP15 heterodimers, indicating the pathogenicity of S428T variant. However, our study was discordant with the previous study [39, 40], for we found 8 women carrying heterozygous S428T variant had a normal phenotype, indicative of a recessive mode of inheritance. It was further confirmed by mouse models, with *Gdf9*^*S415T/WT*^ mice had normal fertility, while *Gdf9*^*S415T/S415T*^ mice showed reduced fecundity and impaired follicular development.

GDF9 plays a pivotal role in the interaction between oocytes and follicle cells. Previous studies on GDF9 at the antral follicle stage were limited since follicular development in *Gdf9* knockout mice ceased at the primary stage. The mouse models established in our study, in which follicles were able to develop to the antral stage, provided a unique opportunity to study the function of oocyte-secreted GDF9 on follicle development at the antral follicle stage.

Previous *in vitro* study demonstrated that GDF9 inhibited 8-Br-cAMP-stimulated STAR expression in both human primary granulosa cells and theca cells*,* resulting in reduced progesterone, 17-hydroxyprogesterone, and DHEA production [16]. In our study, we identified earlier expression of STAR on the small antral follicles in *Gdf9*^*Q308X/S415T*^ mice, indicating GDF9 bi-allelic variants might bring estrogen secretion forward in small antral follicles by relieving its inhibition on STAR expression. Therefore, we proposed that oocyte itself may affect secretion of estrogen from small antral follicles.

GDF9 promotes the proliferation of granulosa cells, as well as DNA synthesis, which was confirmed in previous *in vitro* experiments [14]. As the antrum of the antral follicle gradually enlarges, granulosa cells are divided into cumulus cells which directly surround the oocyte, and mural granulosa cells which are the components of the follicular wall. Oocyte-secreted factors, including GDF9, regulate the differentiation of cumulus cells [41]. While both cumulus cells and mural granulosa cells differentiate from the same common progenitor during folliculogenesis, they exert different functions [41]. Cumulus cells facilitate oocyte maturation and ovulation, whereas mural granulosa cells are mainly responsible for steroid production and differentiation toward luteal cells [41]. From physiological point of view, *GDF9* variants may affect the function of cumulus cells and mural granulosa cells differently. We found that both mural granulosa cell layer and cumulus cell layer were significantly thinner in *Gdf9*^*Q308X/S415T*^ mice compared with wild-type mice. It indicates that GDF9 mutant sequences affect both cumulus cells and mural granulosa cells. However, we did observe differences in these two cell types. The expressions of ki67 and P4HA2 were decreased in mural granulosa cells of *Gdf9*^*Q308X/S415T*^ mice, whereas there was no significant change in cumulus cells. It is hard to explain the difference based on the classic action of GDF9 *via* its receptors. Both cumulus cells and granulosa cells express GDF9 receptors, including ALK5 and BMPR2 [42–44]. No significant difference in the expression levels of BMPR2 in human cumulus cells and mural granulosa cells were detected in previous studies [42]. The expression levels of ALK5 in these two types of cells remain unknown. We speculate that physical distances between these two types of granulosa cells and the oocyte may result in different effects of GDF9 on these two type of cells. Mural granulosa cells may be more affected when the oocyte secretes inadequate GDF9, since they have larger distance from the oocyte compared with cumulus cells. In addition, differential expressed genes in each cell types may be affected by *GDF9* variants in varying degrees, contributing to lower expression of ki67 and P4HA2 in mural granulosa cells but no change in cumulus cells.

Furthermore, *Gdf9*^*S415T/S415T*^ mice presented an intermediate proliferation phenotype between wild-type and *Gdf9*^*Q308X/S415T*^ mice. As a result, we proposed a dose effect of GDF9 on follicular development. Besides, GDF9 affected glycolytic activity of granulosa cells. Decreased glycolytic capacity of granulosa cells may lead to an inadequate energy supply to oocytes [45], which in turn led to poor oocyte quality.

The mechanism of defective follicle enlargement during stimulation remains elusive. We speculated the key issue may be due to the defect in follicular fluid formation. In our transcriptome analysis of granulosa cells, *Has2*, *P4ha2*, *Cldn15,* and *Aqp5* were down-regulated in *Gdf9*^*Q308X/S415T*^ mice. Our study demonstrated that GDF9 promoted Prolyl 4-Hydroxylase Subunit Alpha 2 (*P4HA2*) expression in granulosa cells. P4HA2 is a key enzyme in collagen post-translational modification, which ensures efficient prolyl hydroxylation of collagen, leading to stable triple-helix formation [46]. Down-regulation of *P4ha2* may lead to increased collagen degradation, affecting the osmotic pressure formation in the follicular antrum. Besides, *Has2* is involved in the synthesis of hyaluronic acid, an important extracellular matrix component of follicular fluid, responsible for osmotic pressure formation and paracellular water transport [31]. *Cldn15* encodes one of the tight junction proteins. Tight junctions between granulosa cells limit the escape of macromolecules from the follicular antrum, which helps maintain osmotic pressure [31]. AQP5, a member of the aquaporin family, functions as a water transcellular transporter and may be involved in follicular fluid formation [31, 47]. Meanwhile, down-regulation of *P4ha2* and *Cldn15* can lead to degradation of the extracellular matrix and weakened of cellular junctions, which in turn explain the loosen cumulus cells in *Gdf9*^*Q308X/S415T*^ mice in somehow. Taken together, GDF9 defects may contribute to abnormal antral follicle enlargement by impairing the regulation of various genes involved in follicular fluid formation.

The findings of our study may deepen our understanding of oocyte-derived factors on follicle development. Aged women are characterized by the smaller size of the dominant follicle [48] and early rise of estrogen at the small antral follicle stage [49]. Classically, this phenomenon was explained by decreased inhibin B level which may lead to early elevation of FSH at the antral follicle stage, causing an early rise in estrogen [49]. Since ovarian GDF9 gradually decreased with increasing age [50, 51], we highlighted that oocyte-derived factors, such as GDF9, are implicated in small dominant follicle development in aged women. In addition, unsynchronized follicle development is a commonly seen phenomenon in human ovarian stimulation despite adequate Gn dosage is given, especially in aged women. Our study implied that oocyte itself may control the follicle response in some degree, with poor oocytes in less developed follicles.

The *GDF9*^*Q321X/S428T*^ bi-allelic variant reported in our study resulted in atypical endometrial hyperplasia in human and mouse endometrium, which may be due to prolonged antral follicle development and prolonged exposure to high levels of estrogen. Risk factors for atypical endometrial hyperplasia include obesity, chronic anovulation diseases such as PCOS, and certain genetic factors such as pathogenic variants in DNA mismatch repair genes (*MLH1*, *MSH2*, *MSH6*, *PMS2*, and *EPCAM*) leading to Lynch Syndrome, Cowden Syndrome (*PTEN* pathogenic variant), and Peutz-Jeghers (*STK11* pathogenic variant) [52]. We demonstrated that *GDF9*^*Q321X/S428T*^ variants can lead to atypical endometrial hyperplasia, which increases our understanding of the genetic factors behind atypical endometrial hyperplasia.

However, we did observe heterogeneity in GDF9 biallelic variants. Although similar, phenotypes of individual P1 and P2 were not the same. Younger sister of individual P2, who carried the same GDF9 variants, had a natural pregnancy and delivery at age 27, which was out of our expectation. However, one *Gdf9*^*Q321X/S428T*^ mouse was pregnant at the end of the fertility test, suggesting that the *Gdf9*^*Q321X/S428T*^ variant results in extremely low fertility, but not sterility. We suspected that age effect combined with dose effect of GDF9 exacerbated the follicle maldevelopment in Individual P1 and P2. Therefore, early planning for pregnancy should be advised for women carrying these pathogenic GDF9 variants. In addition, it was possible that many unknown factors, such as paracrine factors and polymorphism of genes responsible for ovarian reserve and/or *GDF9* gene may affect carrier’s phenotype. It is possible other genes regulating GDF9 may contribute to the different phenotype too. For example, *GDF9* was one of the most down-regulated genes in oocytes from individuals carrying a homozygous *TBPL2* variant. Previous studied identified that variants in *TBPL2* caused oocyte maturation defects [53] and oocyte maturation defects [54], leading to female infertility. In recent years, digenic diseases, which are caused by the co-inheritance of DNA variants at two different genetic loci, have been gradually recognized [55–59]. Whether follicular development and female infertility are also affected by digenic inheritance needs to be further explored. Future whole-genome sequencing of P2’s younger sister and comparisons between sequencing data of P2 and her younger sister will be needed to explain the phenotypic inconsistency.

There are some limitations in this study. Firstly, only two *GDF9* biallelic variants were studied in this research, and whether they are representative of other *GDF9* biallelic variants needs to be further investigated. Most of the *GDF9* variants reported in previous studies were heterozygous, with only one homozygous variant. No *GDF9* biallelic variants have been reported before, so we were unable to perform a comparative analysis. However, we hypothesized that the effect of *GDF9* biallelic variants on the reproductive phenotype is related to the effect of the variant itself on the function of the GDF9 protein, for example, the effect of the variant on the GDF9 protein expression, secretory capacity, dimer-forming capacity, and receptor-binding capacity. If a variant results in enhanced function of the GDF9 protein, then it may cause a phenotype inconsistent with the present study. Secondly, our study of individual P2 was retrospective and did not involve collection of follicular fluid or photographs of oocytes. Thirdly, whole-genome sequencing for P2’s sister was not available, therefore, we cannot explain the phenotypic inconsistency between P2 and her sister. Moreover, we regret that we did not detect the genotypes of the control women from whom we collected follicular fluid. However, they all had normal ovarian response with 11days of average Gn duration.

## Conclusion

In conclusion, our study demonstrated that bi-allelic variants in GDF9 cause proliferative defects, abnormal steroidogenesis, and impaired follicular fluid production, which consequently leads to defective follicle development. These findings highlight the essential and conserved role of oocyte-derived GDF9 in estrogen secretion and antral follicular development.

### Supplementary Information

**Additional file 1: Fig. S1.** Pictures of the pregnant *Gdf9*^*Q308X/S415T*^ female. A All the mice were executed and dissected at the end of fertility assessment. One *Gdf9*^*Q308X/S415T*^ female was found pregnant with a near-mature fetus. B Picture of the fetus.

**Additional file 2: Fig. S2.** Effects of heterozygous *Gdf9* variants on fertility. A Average number of pups produced per cage (each cage contains 2 females and 1 male) by females *Gdf9*^*wt/wt*^ (*n*=3), *Gdf9*^*Q308X/wt*^ (*n*=3), and *Gdf9*^*S415T/wt*^ (*n*=3) when paired with wild-type males. B Average litter sizes of* Gdf9*^*wt/wt*^ (*n*=9) , *Gdf9*^*Q308X/wt*^ (*n*=10), and *Gdf9*^*S415T/wt*^ (*n*=11)when paired with wild-type males.

**Additional file 3: Fig. S3.** Effects of *Gdf9* variants on estrous cycle. A Diagrams representing estrous cycles monitored daily over 12 days (M: metestrus, D: diestrus, P: proestrus, and E: estrus). B Days spent in each stage of estrous cycle. *P < 0.05.

**Additional file 4: Fig. S4.** Negative control of immunohistochemistry and immunofluorescence experiments. Expression levels of P4HA2 in human granulosa cells at different stages of follicles analyzed using data from the GEO database (GSE107746). Gene abundance was represented by FPKM.

**Additional file 5: Fig. S5.** Negative control of immunohistochemistry and immunofluorescence experiments. A Negative control of ki67 immunohistochemistry experiment. B Negative control of P4HA2 immunohistochemistry experiment. C Negative control of TUNEL immunofluorescence experiment. Nuclei were stained with DAPI (the excitation wavelength was 364nm). Dead cells were stained with TMR red (the excitation wavelength was 555nm). The dotted lines outline the boundaries of granulosa cells. D Negative control of STAR immunofluorescence experiment. Nuclei were stained with DAPI (the excitation wavelength was 364nm). The secondary antibody used was Alexa Fluor 647 goat anti-rabbit IgG (the excitation wavelength was 647nm). The dotted lines outline the boundaries of granulosa cells.

**Additional file 6: Fig. S6.** Low magnification images of immunofluorescence experiments. A Low magnification images of TUNEL immunofluorescence experiment. TUNEL (red) and DAPI (blue) immunofluorescence in *Gdf9*^*wt/wt*^, *Gdf9*^*S415T/S415T*^ and *Gdf9*^*Q308X/S415T*^ ovaries. The dotted lines outline the boundaries of granulosa cells. B Low magnification images of STAR immunofluorescence experiment. STAR (yellow) and DAPI (blue) immunofluorescence in *Gdf9*^*wt/wt*^ and *Gdf9*^*Q308X/S415T*^ ovaries. The dotted lines outline the boundaries of granulosa cells.

**Additional file 7: Fig. S7.** Original and full-length blot images. A Uncropped blot images corresponding to Fig.3A. B Uncropped blot images corresponding to Fig.3C. C Uncropped blot images corresponding to Fig.3D. D Uncropped blot images corresponding to Fig.3E. E Uncropped blot images corresponding to Fig.3G. F Uncropped blot images corresponding to Fig.3F. G Uncropped blot images corresponding to Fig.7H.

**Additional file 8: Table S1. **List of sgRNAs and human or mouse genomic PCR primers. Tm: Melting Temperature.

**Additional file 9: Table S2. **Clinical characteristics and IVF/ICSI outcomes of controls with normal ovarian response.

**Additional file 10: Table S3. **Clinical characteristics and IVF/ICSI outcomes of individuals with heterozygous *GDF9*^*S428T*^ variant.

**Additional file 11: Table S4. **Enrichment analysis of GO-BP (Gene Ontology-Biological Process) using GSEA method.

**Additional file 12: Table S5. **Enrichment analysis of Reactome (obtained from MiSigDB) using GSEA method.

## Data Availability

The datasets used and/or analysed during the current study are available from the corresponding author on reasonable request.

## Abbreviations

WES: Whole-exome sequencing
ART: Assisted reproductive technology
COS: Controlled ovarian stimulation
POR: Poor ovarian response
Gn: Gonadotropin
GDF: Growth differentiation factor
POI: Primary ovarian insufficiency
hGCs: Human primary luteinized granulosa cells
HCG: Human chorionic gonadotropin
COC: Cumulus-oocyte complexs
PBS: Phosphate-buffered saline
GO-BP: Gene Ontology- Biological Process
