# Supplementary figures and images for: GDF9His209GlnfsTer6/S428T and GDF9Q321X/S428T bi-allelic variants caused female subfertility with defective follicle enlargement
Source: Cell Commun Signal. 2024 Apr 20;22:235. doi: 10.1186/s12964-024-01616-8 (PMC11031944; doi:10.1186/s12964-024-01616-8)

**A**

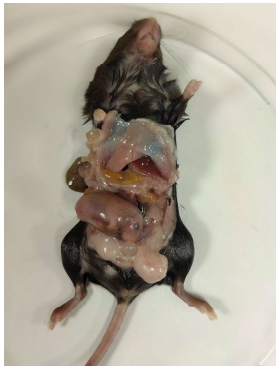

**B**

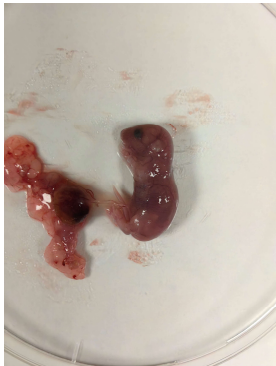

Supplement: Supplementary file 1 — Additional file 1: Fig. S1. Pictures of the pregnant Gdf9Q308X/S415T female. A All the mice were executed and dissected at the end of fertility assessment. One Gdf9Q308X/S415T female was found pregnant with a near-mature fetus. B Picture of the fetus. [file 12964_2024_1616_MOESM1_ESM.pdf]

# A

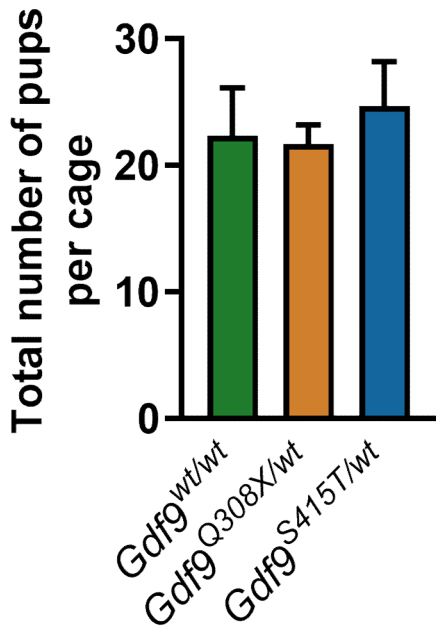

# B

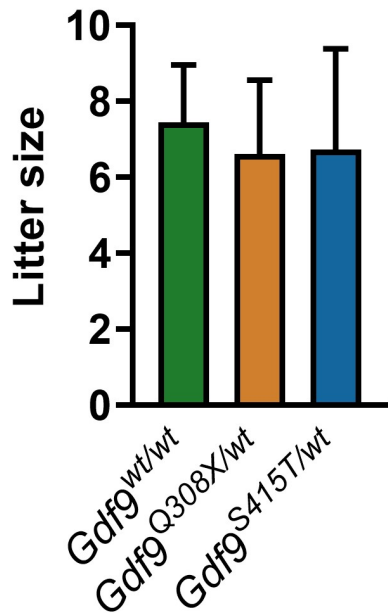

Supplement: Supplementary file 2 — Additional file 2: Fig. S2. Effects of heterozygous Gdf9 variants on fertility. A Average number of pups produced per cage (each cage contains 2 females and 1 male) by females Gdf9wt/wt (n=3), Gdf9Q308X/wt (n=3), and Gdf9S415T/wt (n=3) when paired with wild-type males. B Average litter sizes of Gdf9wt/wt (n=9) , Gdf9Q308X/wt (n=10), and Gdf9S415T/wt (n=11)when paired with wild-type males. [file 12964_2024_1616_MOESM2_ESM.pdf]

**A**

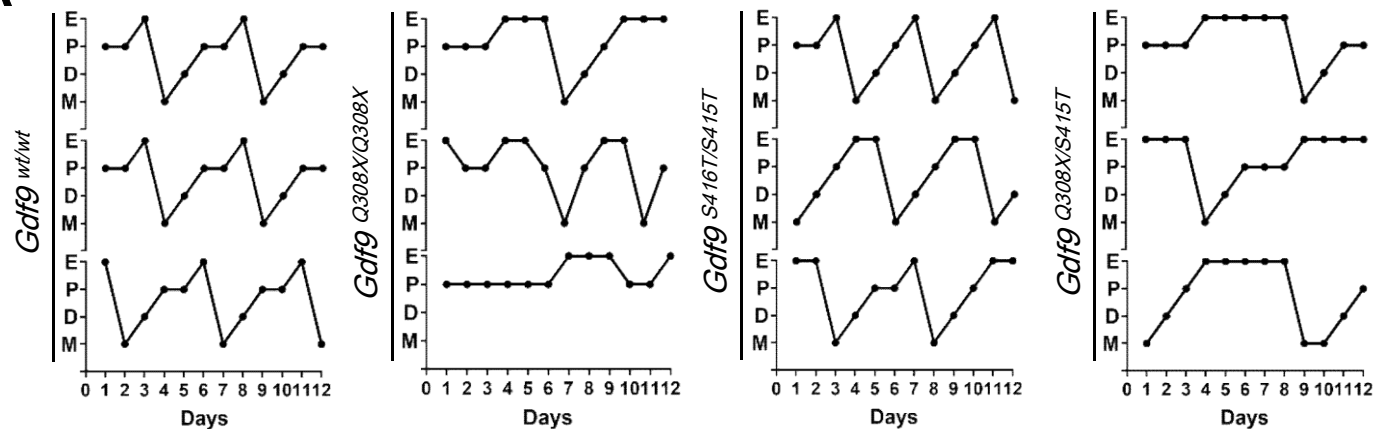

**B**

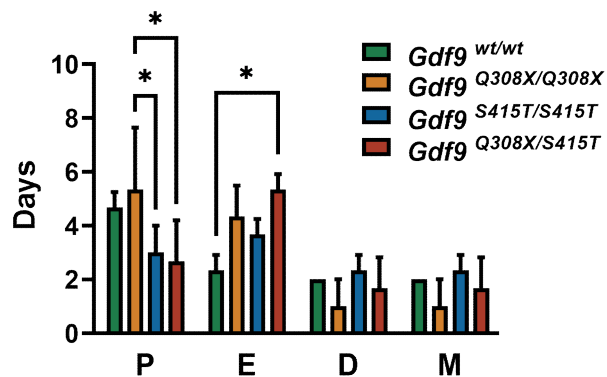

Supplement: Supplementary file 3 — Additional file 3: Fig. S3. Effects of Gdf9 variants on estrous cycle. A Diagrams representing estrous cycles monitored daily over 12 days (M: metestrus, D: diestrus, P: proestrus, and E: estrus). B Days spent in each stage of estrous cycle. *P < 0.05. [file 12964_2024_1616_MOESM3_ESM.pdf]

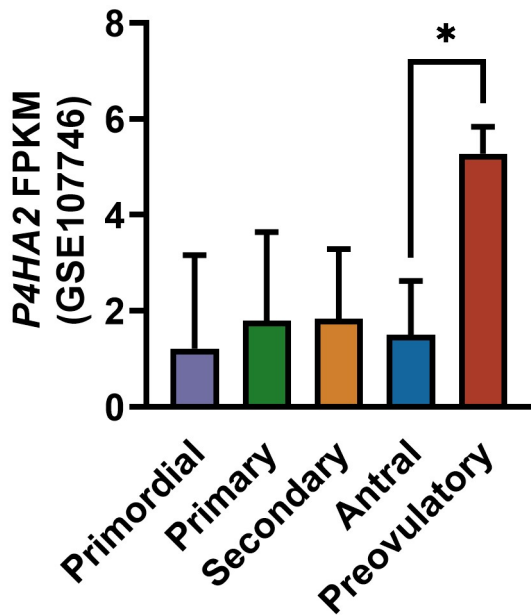

Supplement: Supplementary file 4 — Additional file 4: Fig. S4. Negative control of immunohistochemistry and immunofluorescence experiments. Expression levels of P4HA2 in human granulosa cells at different stages of follicles analyzed using data from the GEO database (GSE107746). Gene abundance was represented by FPKM. [file 12964_2024_1616_MOESM4_ESM.pdf]

A

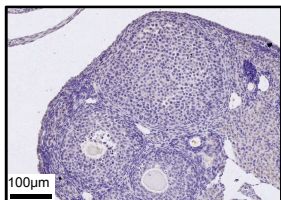

B

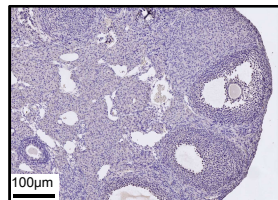

C

DAPI

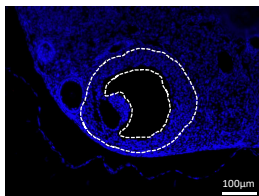

555nm

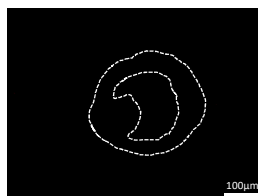

Merge

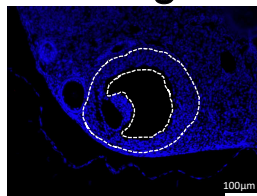

D

DAPI

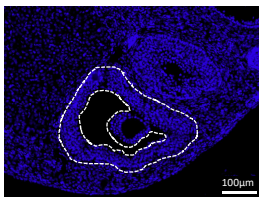

647nm

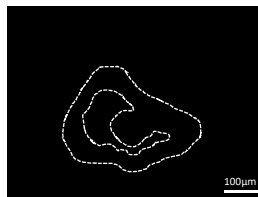

Merge

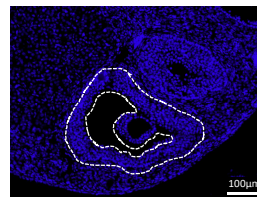

Supplement: Supplementary file 5 — Additional file 5: Fig. S5. Negative control of immunohistochemistry and immunofluorescence experiments. A Negative control of ki67 immunohistochemistry experiment. B Negative control of P4HA2 immunohistochemistry experiment. C Negative control of TUNEL immunofluorescence experiment. Nuclei were stained with DAPI (the excitation wavelength was 364nm). Dead cells were stained with TMR red (the excitation wavelength was 555nm). The dotted lines outline the boundaries of granulosa cells. D Negative control of STAR immunofluorescence experiment. Nuclei were stained with DAPI (the excitation wavelength was 364nm). The secondary antibody used was Alexa Fluor 647 goat anti-rabbit IgG (the excitation wavelength was 647nm). The dotted lines outline the boundaries of granulosa cells. [file 12964_2024_1616_MOESM5_ESM.pdf]

A

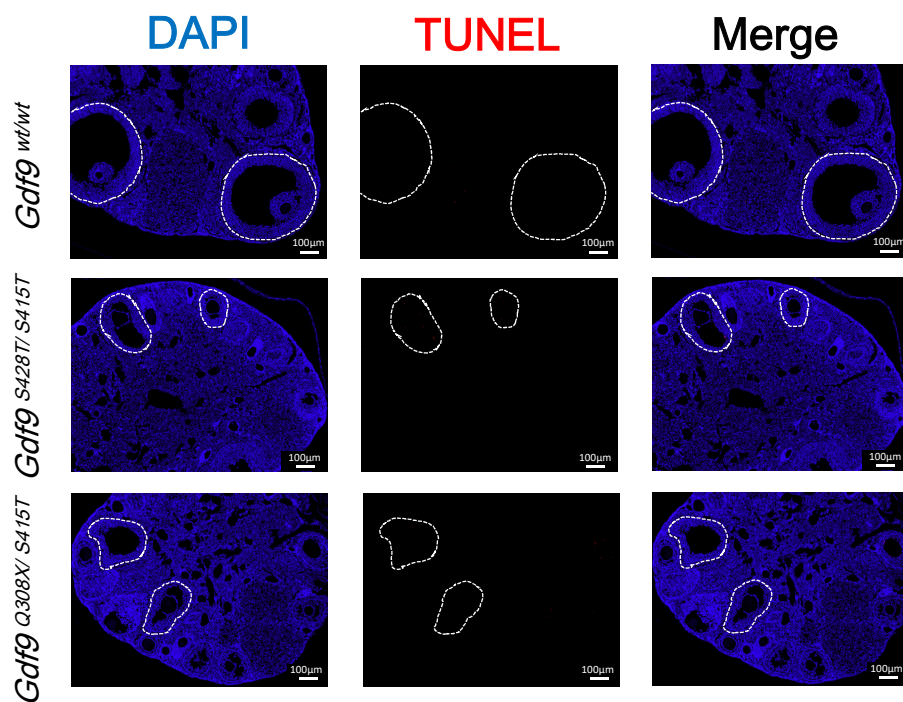

B

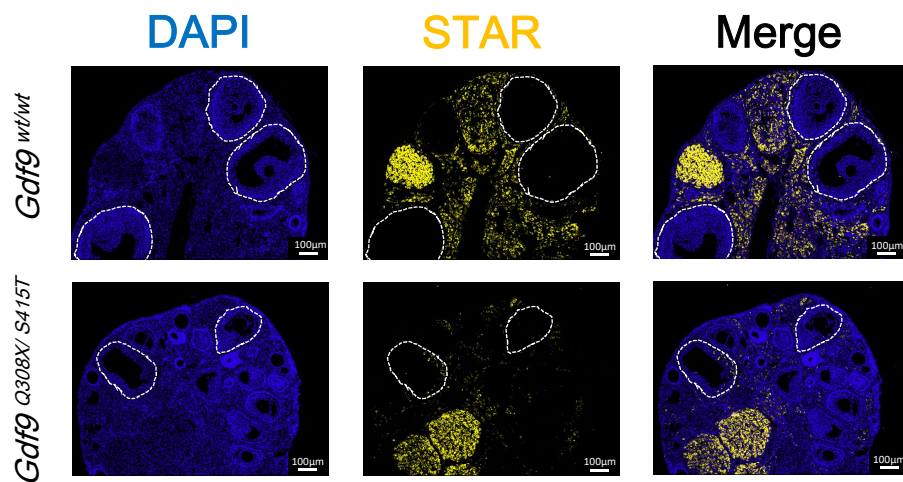

Supplement: Supplementary file 6 — Additional file 6: Fig. S6. Low magnification images of immunofluorescence experiments. A Low magnification images of TUNEL immunofluorescence experiment. TUNEL (red) and DAPI (blue) immunofluorescence in Gdf9wt/wt, Gdf9S415T/S415T and Gdf9Q308X/S415T ovaries. The dotted lines outline the boundaries of granulosa cells. B Low magnification images of STAR immunofluorescence experiment. STAR (yellow) and DAPI (blue) immunofluorescence in Gdf9wt/wt and Gdf9Q308X/S415T ovaries. The dotted lines outline the boundaries of granulosa cells. [file 12964_2024_1616_MOESM6_ESM.pdf]

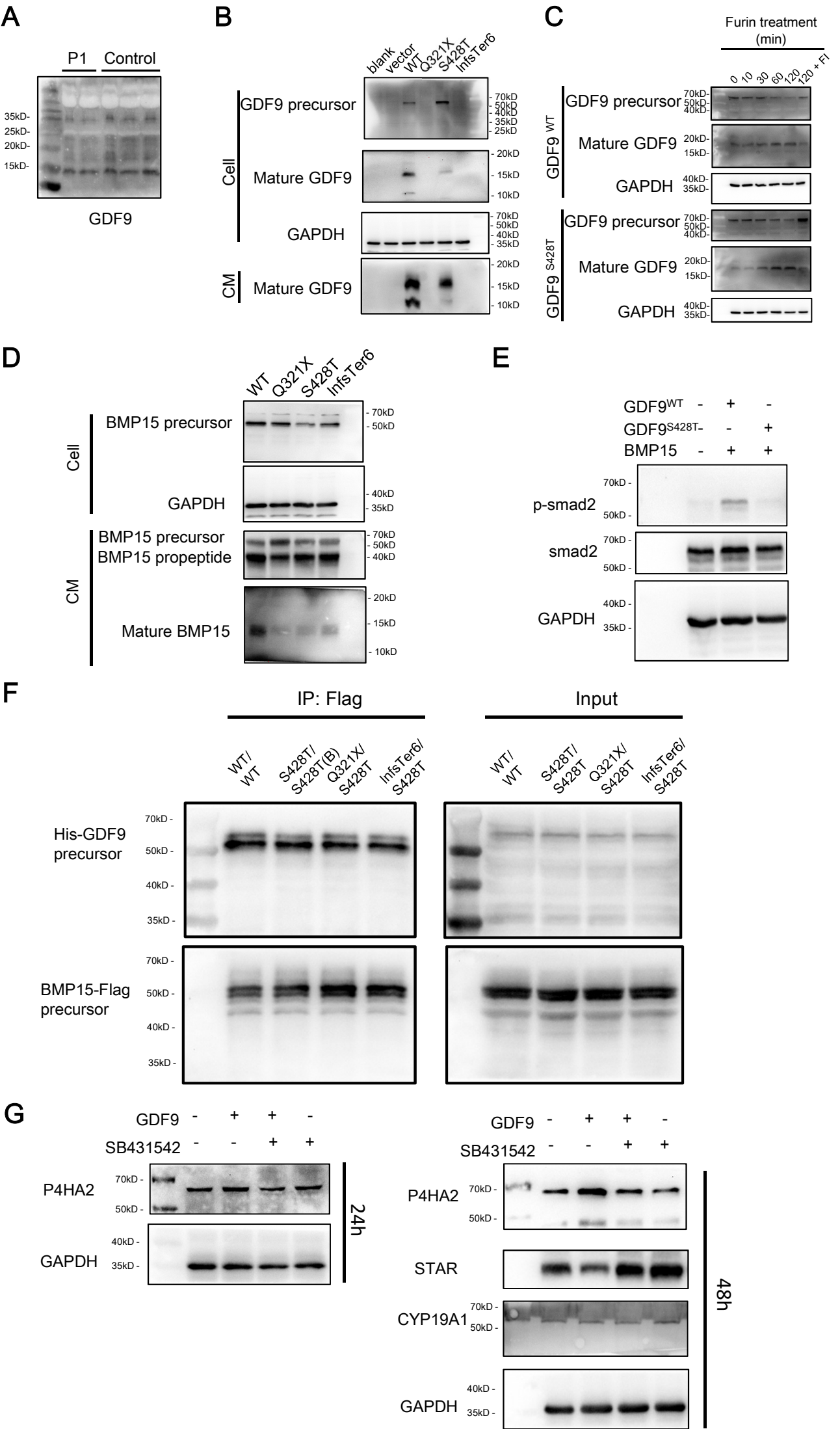

Supplement: Supplementary file 7 — Additional file 7: Fig. S7. Original and full-length blot images. A Uncropped blot images corresponding to Fig.3A. B Uncropped blot images corresponding to Fig.3C. C Uncropped blot images corresponding to Fig.3D. D Uncropped blot images corresponding to Fig.3E. E Uncropped blot images corresponding to Fig.3G. F Uncropped blot images corresponding to Fig.3F. G Uncropped blot images corresponding to Fig.7H. [file 12964_2024_1616_MOESM7_ESM.pdf]
