# Supplementary material for: GDF9His209GlnfsTer6/S428T and GDF9Q321X/S428T bi-allelic variants caused female subfertility with defective follicle enlargement
Source: Cell Commun Signal. 2024 Apr 20;22:235. doi: 10.1186/s12964-024-01616-8 (PMC11031944; doi:10.1186/s12964-024-01616-8)
Supplement: Supplementary file 8 — Additional file 8: Table S1. List of sgRNAs and human or mouse genomic PCR primers. Tm: Melting Temperature. [file 12964_2024_1616_MOESM8_ESM.docx]

**Table S1. List of sgRNAs and human or mouse genomic PCR primers**

| Oligo | Sequence (5’ to 3’) | Type | Tm  (℃) | Product size (bp) | Application |
| --- | --- | --- | --- | --- | --- |
| sgRNA-Q308X | GGATGGCTTTCTGCCCTCGA | sgRNA | / | / | sgRNAs for *Gdf9* knock-in mouse lines |
| sgRNA-S415T | GTTCAATGGTCAACACACTC | sgRNA | / | / |  |
| hGDF9-Q321X-F | CCTTCAGCACAGAATGGTTTG | Primer | 56.9 | 423 | Primers for Sanger sequencing of human variants |
| hGDF9- Q321X -R | TTGGACAGTCCCCTTTACAGTATC | Primer | 57.3 |  |  |
| hGDF9-S428T-F | TGAAGCTTTCTCTTGAAGGCACACA | Primer | 63.2 | 512 |  |
| hGDF9-S428T-R | CTTCCCATCACCGTCACCG | Primer | 60.9 |  |  |
| hGDF9-His209GInfsTer6-F | CCAGTTGTCCCACTTCAGCTGACTA | Primer | 62.6 | 548 |  |
| hGDF9- His209GInfsTer6-R | GGACTCTCGGCAGAGCTCCA | Primer | 61.6 |  |  |
| mGdf9-Q308X-F | CAGAGGACGGAGTGTTTAGCATG | Primer | 59.8 | 491 | Primers for Sanger sequencing of mouse variants |
| mGdf9-Q308X-R | CACTGAAGGGTCCAGCTTCTCATA | Primer | 60.6 |  |  |
| mGdf9-S415T-F | TCAAATGGGACAACTGGATCGTG | Primer | 62.8 | 346 |  |
| mGdf9-S415T-R | AAGCTCTCTGCCCAGGCACTCTAA | Primer | 63.6 |  |  |
| mFshr-F | CCTTGCTCCTGGTCTCCTTG | Primer | 58.5 | 113 | Primers for real-time RT-PCR |
| mFshr-R | CTCGGTCACCTTGCTATCTTG | Primer | 56.2 |  |  |
| mLhcgr-F | TTCCAAGGGATGAATAACGAGTCT | Primer | 59.0 | 75 |  |
| mLhcgr-R | TGCATGGCTTTGTACTTCTTCAA | Primer | 58.6 |  |  |
| mStar-F | AAAGGCCTTGGGCATACTCA | Primer | 58.3 | 88 |  |
| mStar-R | GCACCACCTTACTTAGCACTTCATCT | Primer | 60.1 |  |  |
| mCyp11a1-F | GTCCCACTCCTCAAAGCCAG | Primer | 58.3 | 94 |  |
| mCyp11a1-R | GAAGCACCAGGTCGTTCACAAT | Primer | 60.0 |  |  |
| mCyp17a1-F | GCCCAAGTCAAAGACACCTAAT | Primer | 56.1 | 159 |  |
| mCyp17a1-R | GTACCCAGGCGAAGAGAATAGA | Primer | 56.3 |  |  |
| mHsd3b2-F | CCAGGGCATCTCTGTTGTCAT | Primer | 58.6 | 104 |  |
| mHsd3b2-R | GGTTCTGGGTACCTTTCAGATTGA | Primer | 59.4 |  |  |
| mCyp19a1-F | ATGTTCTTGGAAATGCTGAACCC | Primer | 60.2 | 150 |  |
| mCyp19a1-R | AGGACCTGGTATTGAAGACGAG | Primer | 56.1 |  |  |
| mP4ha2-F | CACCTCCATTGGGCACATGA | Primer | 61.3 | 109 |  |
| mP4ha2-R | GCTCTTAATCTTGGCGAGCTT | Primer | 56.7 |  |  |
| mGapdh-F | AACCTGCCAAGTATGATGACATCA | Primer | 59.4 | 111 |  |
| mGapdh-R | TGTTGAAGTCACAGGAGACAACCT | Primer | 58.8 |  |  |
| hP4HA2-F | CAAACTGGTGAAGCGGCTAAA | Primer | 59.0 | 95 |  |
| hP4HA2-R | GCACAGAGAGGTTGGCGATA | Primer | 57.5 |  |  |
| hGAPDH-F | GCACCGTCAAGGCTGAGAAC | Primer | 59.4 | 138 |  |
| hGAPDH-R | TGGTGAAGACGCCAGTGGA | Primer | 59.8 |  |  |
