# Supplementary material for: GDF9His209GlnfsTer6/S428T and GDF9Q321X/S428T bi-allelic variants caused female subfertility with defective follicle enlargement
Source: Cell Commun Signal. 2024 Apr 20;22:235. doi: 10.1186/s12964-024-01616-8 (PMC11031944; doi:10.1186/s12964-024-01616-8)
Supplement: Supplementary file 9 — Additional file 9: Table S2. Clinical characteristics and IVF/ICSI outcomes of controls with normal ovarian response. [file 12964_2024_1616_MOESM9_ESM.docx]

**Table S2. Clinical characteristics and IVF/ICSI outcomes of controls with normal ovarian response**

|  | C1 | C2 | C3 | C4 | C5 | C6 |
| --- | --- | --- | --- | --- | --- | --- |
| Age (years) | 36 | 36 | 36 | 36 | 36 | 36 |
| BMI (kg/m^2^) | 18.1 | 20.1 | 22.4 | 22.4 | 21.8 | 17.3 |
| Menstrual cycle (Days) | 25 | 30 | 27-28 | 34 | 25 | 26-28 |
| FSH/LH (mIU/ml) | 10.65/2.94 | 6.98/5.13 | 4.81/2.45 | 4.53/1.36 | 10.4/2.96 | 7.18/3.51 |
| Type of Infertility | Secondary | Secondary | Secondary | Primary | Primary | Secondary |
| Gn starting dose (IU) | 300 | 225 | 300 | 300 | 300 | 300 |
| Days of Gn stimulation | 13 | 10 | 11 | 10 | 10 | 12 |
| Total Gn dosage (IU) | 3900 | 2550 | 3300 | 3000 | 2850 | 3600 |
| E_2_ level on the day of hCG injection (pg/ml) | 1741 | 3970 | 2038 | 2193 | 1102 | 2056 |
| Number of follicles at hCG injection |  |  |  |  |  |  |
| d* ≥18 | 3 | 1 | 4 | 4 | 1 | 5 |
| 15 ≤ d <18 | 1 | 5 | 3 | 4 | 0 | 1 |
| 10 ≤ d <15 | 4 | 9 | 4 | 6 | 4 | 2 |
| 5 ≤ d <10 | 0 | 5 | 0 | 0 | 2 | 1 |
| Retrieved oocytes | 6 | 15 | 11 | 11 | 4 | 6 |
| Fertilization method | IVF | IVF | IVF | IVF | ICSI | IVF |
| Fertilization rate (2PN) | 6/6 | 6/15 | 7/11 | 7/11 | 1/4 | 5/6 |
| High-quality embryo rate (D3) | 4/11 | 6/13 | 3/6 | 6/13 | 8/10 | 4/12 |
| Clinical pregnancy (cumulative outcome) | Yes | Yes | Yes | Yes | Yes | Yes |
| Live birth (cumulative outcome) | Yes | Yes | Yes | Yes | Yes | Yes |

C1-8: Representing control women with normal ovarian response. IVF: *in vitro* fertilization; ICSI: intracytoplasmic sperm injection. *d= diameters.
