# Supplementary material for: GDF9His209GlnfsTer6/S428T and GDF9Q321X/S428T bi-allelic variants caused female subfertility with defective follicle enlargement
Source: Cell Commun Signal. 2024 Apr 20;22:235. doi: 10.1186/s12964-024-01616-8 (PMC11031944; doi:10.1186/s12964-024-01616-8)
Supplement: Supplementary file 10 — Additional file 10: Table S3. Clinical characteristics and IVF/ICSI outcomes of individuals with heterozygous GDF9S428T variant. [file 12964_2024_1616_MOESM10_ESM.docx]

**Table S3. Clinical characteristics and IVF/ICSI outcomes of individuals with heterozygous *GDF9^S428T^* variant**

|  | H1 | H2 | H3 | H4 | H5 | H6 | H7 | H8 |
| --- | --- | --- | --- | --- | --- | --- | --- | --- |
| Age (years) | 36 | 35 | 27 | 31 | 29 | 26 | 28 | 36 |
| BMI (kg/m^2^) | 30.0 | 26.6 | 20.1 | 19.1 | 24.4 | 20.4 | 20.2 | 22.8 |
| Menstrual cycle (Days) | 30 | 27-28 | 28 | 30-31 | 30 | 28-35 | 33-34 | 28 |
| FSH/LH (mIU/ml) | 5.89/2.98 | 7.11/3.15 | 9.94/5.01 | 5.54/3.85 | 6.67/3.94 | 5.44/3.78 | 3.51/2.3 | 4.78/1.96 |
| AFC (bilateral) | 20 | 14 | 12 | 15 | 10 | 19 | 14 | 13 |
| Type of Infertility | Primary | Primary | Secondary | Secondary | Primary | Primary | Secondary | Primary |
| Gn starting dose (IU) | 225 | 200 | 225 | 225 | 300 | 137.5 | 150 | 250 |
| Days of Gn stimulation | 12 | 11 | 12 | 10 | 9 | 11 | 11 | 12 |
| Total Gn dosage (IU) | 2925 | 2250 | 3075 | 2400 | 2700 | 1600 | 1575 | 3000 |
| E_2_ level on the day of hCG injection (pg/ml) | >5000 | 2634 | 1736 | 2882 | 1406 | 2415 | 2697 | 1670 |
| Number of follicles at hCG injection |  |  |  |  |  |  |  |  |
| d* ≥18 | 6 | 2 | 2 | 3 | 4 | 2 | 3 | 5 |
| 15 ≤ d <18 | 10 | 6 | 3 | 4 | 0 | 5 | 3 | 2 |
| 10 ≤ d <15 | 3 | 5 | 5 | 7 | 3 | 5 | 6 | 5 |
| 5 ≤ d <10 | 2 | 3 | 2 | 1 | 1 | 1 | 3 | 1 |
| Retrieved oocytes | 28 | 14 | 8 | 13 | 11 | 16 | 17 | 9 |
| Fertilization method | ICSI | IVF | IVF | IVF | ICSI | ICSI | IVF | ICSI |
| Fertilization rate (2PN) | 11/22 | 13/14 | 6/8 | 13/13 | 10/10 | 12/12 | 13/17 | 7/9 |
| High-quality embryo rate (D3) | 4/11 | 6/13 | 3/6 | 6/13 | 8/10 | 4/12 | 2/13 | 3/7 |
| Clinical pregnancy (cumulative outcome) | Yes | Yes | Yes | Yes | Yes | Yes | No | No |
| Live birth (cumulative outcome) | Yes | Yes | Yes | Yes | Yes | Yes | No | No |

H1-8: Representing infertile women carrying heterozygous *GDF9^S428T^* variant; IVF: *in vitro* fertilization; ICSI: intracytoplasmic sperm injection. *d= diameters.
